# Spastin mutations impair coordination between lipid droplet dispersion and reticulum

**DOI:** 10.1371/journal.pgen.1008665

**Published:** 2020-04-21

**Authors:** Yoan Arribat, Dogan Grepper, Sylviane Lagarrigue, Timothy Qi, Sarah Cohen, Francesca Amati

**Affiliations:** 1 Aging and Muscle Metabolism Lab, Department of Biomedical Sciences, School of Biology and Medicine, University of Lausanne, Lausanne, Switzerland; 2 Department of Cell Biology and Physiology, The University of North Carolina at Chapel Hill, Chapel Hill, North Carolina, United States; 3 Service of Endocrinology, Diabetology & Metabolism, Lausanne University Hospital and Lausanne University, Lausanne, Switzerland; Institut Jacque Monod, Centre National de la Recherche Scientifique, FRANCE

## Abstract

Lipid droplets (LD) are affected in multiple human disorders. These highly dynamic organelles are involved in many cellular roles. While their intracellular dispersion is crucial to ensure their function and other organelles-contact, underlying mechanisms are still unclear. Here we show that Spastin, one of the major proteins involved in Hereditary Spastic Paraplegia (HSP), controls LD dispersion. Spastin depletion in zebrafish affects metabolic properties and organelle dynamics. These functions are ensured by a conserved complex set of splice variants. M1 isoforms determine LD dispersion in the cell by orchestrating endoplasmic reticulum (ER) shape along microtubules (MTs). To further impact LD fate, Spastin modulates transcripts levels and subcellular location of other HSP key players, notably Seipin and REEP1. In pathological conditions, mutations in human Spastin M1 disrupt this mechanism and impacts LD network. Spastin depletion influences not only other key proteins but also modulates specific neutral lipids and phospholipids, revealing an impact on membrane and organelle components. Altogether our results show that Spastin and its partners converge in a common machinery that coordinates LD dispersion and ER shape along MTs. Any alteration of this system results in HSP clinical features and impacts lipids profile, thus opening new avenues for novel biomarkers of HSP.

## Author summary

Hereditary spastic paraplegia is a group of inherited diseases characterized by spasticity and gait disorder. Spastin mutations represent the most common cause of hereditary spastic paraplegia. While Spastin was initially defined as a microtubule severing protein, alternative isoforms impact organelles. In the present work, we identify how Spastin combines the specificity of different splice variants to coordinate endoplasmic reticulum shape and lipid droplet dispersion. When mutated, Spastin impairs this synchronization between organelle dynamics, and affects other proteins involved in hereditary spastic paraplegia, notably REEP1 and Seipin. The coordination of lipid droplet dispersion and reticulum shaping under the control of Spastin is a unique trafficking model, affected in the different forms of hereditary spastic paraplegia. The impact of Spastin on this machinery results in the alteration of the lipid profile, particularly phospholipids and neutral lipids in muscle and brain. These potential biomarkers open new therapeutic avenues in hereditary spastic paraplegia.

## Introduction

Lipid droplets (LDs) have long been considered as inert organelles, limited to fat storage. However, a wide range of evidence recently highlighted the multiple functions of LDs in energy supply, embryogenesis, reactive oxygen species management or pathogen invasion [1–6]. Present in bacteria, yeast, plants and animals, LDs are not limited to adipose tissue in mammals. LDs play determinant functions in many cells, including glial cells, neurons or myocytes [4, 7]. To face cell-specific needs, LDs adapt in size and number through complex machineries [8], comparable to mitochondrial dynamics. The proteins and mechanisms underlying these pathways are partially elucidated [9]. LD biogenesis starts with the accumulation of neutral lipids in the intermembrane space of the endoplasmic reticulum (ER). Among key proteins, Seipin has been shown to control the budding of nascent LD [10–13]. The nuclear membrane recently appeared as a second source of LD [14]. LDs can adapt their caliber through fusion and fission [15], notably via the role of cell death-inducing DFF45-like effector family proteins [16]. Degradation is supported by two complementary mechanisms: enzymatic lipolysis and lipophagy [17].

LDs are well-connected organelles with multiple inter-organellar communications [8, 18]. Beyond the biogenesis process, mature LDs remain in contact with the ER [19, 20], allowing the transfer of lipids and proteins [21]. Contact sites with mitochondria [22, 23] and peroxisomes [24] enhance fatty acid exchanges and modulate metabolic functions. These inter-organellar interactions suggest the existence of a finely tuned trafficking. The first evidence of LD transport comes from the drosophila model and highlights the importance of the gene *klarsicht* [25]. Further studies identified Halo as a cofactor of Kinesin-1, controlling LD transport along Microtubules (MTs) in drosophila embryos [26]. However, Halo and Klar have no orthologue in vertebrates. The importance of MTs for LD trafficking has been confirmed in mammalian cells, particularly to adapt to nutritional states [27]. In rat liver cells, Kinesin-1 has been shown to be recruited at the LD surface in fed conditions suggesting the existence of active transport of these organelles [28].

The exact processes and the molecular actors that lead to the distribution and spreading of LDs in vertebrate cells are still unknown. Similarly to Miro-1 and Milton, which are required for mitochondria trafficking along MTs [29], proteins involved in LD transport should exhibit targeting to both MTs and LDs. Until now, none of the LD coating proteins exhibit interactions with the cytoskeleton. The identification of Spastin isoform M1 at the LD surface makes it a great candidate [30]. Spastin is an evolutionary conserved protein that exhibits the unique capacity of severing MTs [31–33]. Mutations of the *SPAST* gene are responsible for autosomal dominant cases of Hereditary Spastic Paraplegia (HSP); a group of neurodegenerative disorders affecting upper motor neurons [34, 35]. The canonical isoform of Spastin, referred to as M87 in human, exhibits a strong MT-targeting domain in the N-terminus and an AAA ATPase enzymatic function of the C-terminus. The latter participates in the severing activity [32], which has been extensively described in wild-type and mutant conditions [33, 36]. The existence of an alternative ATG initiator revealed a second transcript encoding for the isoform M1 with a hydrophobic N-terminal domain [37]. This long isoform conserves the MT-targeting domain and strongly binds LD in mammalian cells [30]. Mutations in Spastin isoform M1 have been described to alter axonal transport and intracellular organelles distribution [38–40].

In this report, we propose an extensive description of Spastin splice isoforms, from zebrafish to human. The generation of a CRISPR-Cas9 knockout model supports a role for Spastin in LD dynamics. Spastin deletion affects metabolic properties and organelle characteristics in fish. We further confirm in human cells that Spastin regulates LD formation and dispersion through the reorganization of ER along MTs. Spastin isoforms ensure their function in synergy with other HSP-related proteins such as REEP1 and Seipin. In mutant conditions, Spastin generates a reorganization of ER with a subsequent impact on LDs. Altogether, our results provide evidence that Spastin and other HSP-related proteins synchronize the shaping of ER and MTs stability to determine the dispersion of LDs in the cell, converging in comparable clinical features in case of alteration.

## Results

### Spastin, a complex set of splice isoforms in zebrafish

**Fig 1:**
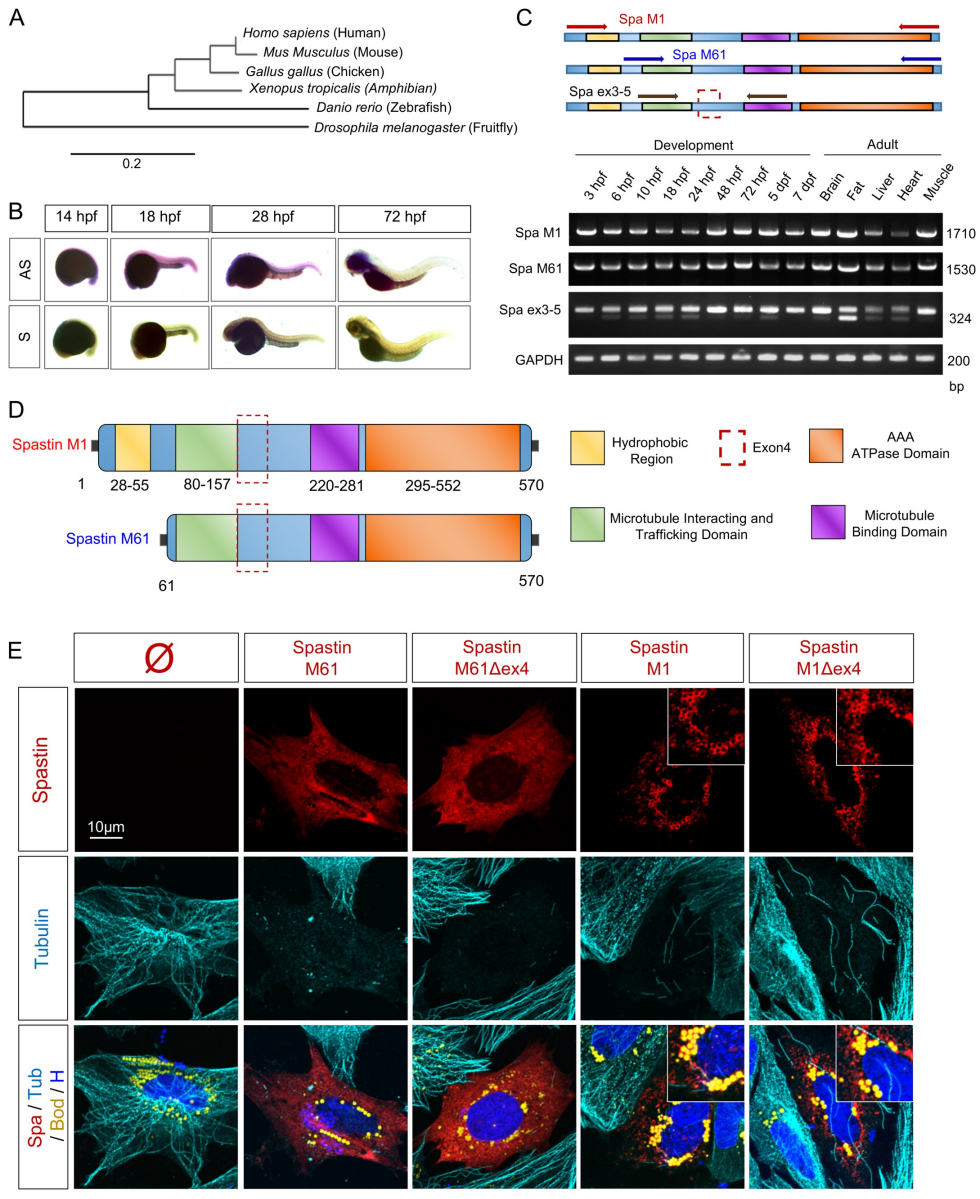
Spastin splice variants in zebrafish. (A) Spastin phylogenic tree (designed from www.phylogeny.fr –Substitution model: Dayhoff). (B) Whole-mount *in situ* hybridization of Spastin transcript in zebrafish embryos (at 14, 18, 28, 72 hpf). Antisense probe (AS) correspond to Spastin transcript. Sense probe (S) to negative control. n = 10 embryos for each group. (C) RT-PCR amplifying different regions of Spastin from embryonic and adult zebrafish cDNA. (D) Schematic representation of Spastin splice variants in zebrafish. (E) Confocal microscopy images of zebrafish embryonic cells overexpressing Spastin splice variants (treated with 300μM oleic acid for 18h before acquisition). Top right insets correspond to higher magnification centered on LDs. Cherry-tagged Spastin appears in red. Tubulin labeling corresponds to microtubules (cyan), Bodipy to LDs (yellow) and Hoechst to nucleus (blue). N = 4 experiments. See also S1 Fig.

Spastin is an ancient protein, putatively present in plants, that exhibits a high conservation of amino-acid identity in mammals [30, 31, 41–43] (Fig 1A). Due to the direct involvement of Spastin (OMIM# 604277) in more than 40% of autosomal dominant HSP (SPG4, OMIM# 182601) [44, 45], its impact on motor neuron and cytoskeleton has been extensively characterized in different animal models [31, 38, 43]. Zebrafish has been shown to be an excellent model to investigate the role of Spastin in motor neuron organization [46, 47]. The conservation of the two initiator sites of the gene has been confirmed in fish [48], suggesting that the different transcripts of Spastin play a key role through evolution. First, to characterize the whole-body expression pattern of Spastin, we performed *in situ* hybridization at key stages of zebrafish embryogenesis (Fig 1B). Antisense probe staining exhibited an ubiquitous expression of Spastin in early stages of development from 14 to 28 hours post-fertilization (hpf) followed by an accumulation of the transcript in the central nervous system at the end of embryogenesis (72 hpf). In parallel, we used RT-PCR to decipher the existence of different transcripts during zebrafish embryogenesis and in the adult (Fig 1C). This approach revealed a stable expression of the short and long transcripts, respectively M61 and M1, throughout embryonic and larval development. In adult fish, Spastin isoforms were ubiquitous but more abundantly expressed in brain, fat and skeletal muscle (Fig 1C). As described by Jardin *et al*. [48], a splice variant affecting exon 4 was detectable with the use of specific primers amplifying exon 3 and 5. This splicing event was particularly strong in fat and during the first half of embryogenesis from 6 to 24 hpf (Fig 1C).

Cloning Spastin isoforms from 24 hpf embryos cDNA confirmed the co-existence of four transcripts. All the resulting Spastin proteins conserved the MT interacting and trafficking domain, the MT binding domain and the c-terminal AAA ATPase domain, but differed by the presence of exon 4 and the initiator methionine (Fig 1D). Expression of the different Spastin variants in zebrafish embryonic cells confirmed that short isoforms M61 and M61Δex4 shared a strong severing activity, as overexpression of these isoforms nearly eliminated the MT network compared to un-transfected cells (Fig 1E). The long isoforms M1 and M1Δex4 targeted LDs (Fig 1E), which is explained by a hydrophobic region generated by the alternative initiator codon (Fig 1D), while also partially conserving the severing property.

Further, we found evidence for a third pair of splice variants lacking a large region from exon 2 to 10 (S1A Fig). Detected both in embryos and adult tissues, these splice variants lost the severing activity but exhibited a strong location in ER and LDs (S1B and S1C Fig). Although alternative isoforms have been described in human Spastin corresponding to the suppression of exon 8 and 15 [49], this splicing event has not yet been reported in mammals which is why we have decided to focus on the variants presented in Fig 1D.

Taken together, these results highlighted the complex role of Spastin, as a function of its transcripts, and confirmed that the zebrafish is a powerful model to investigate the alternative roles of this protein in the whole organism.

### Spastin depletion impacts metabolic properties in knockout animals

**Fig 2:**
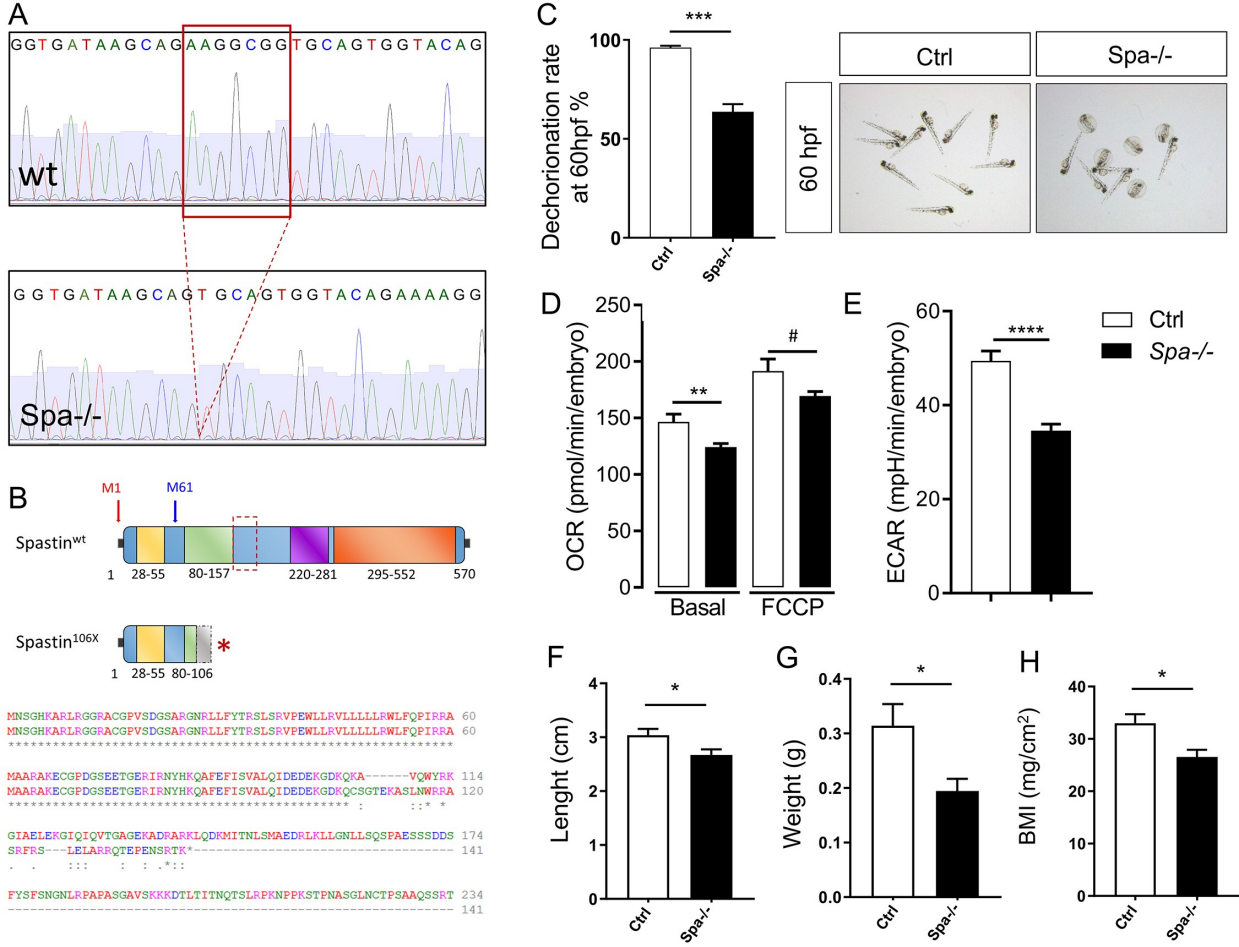
Spastin depletion impairs respiration and growth in zebrafish. (A) Representation of Spastin deletion in CRISPR/Cas9 knockout zebrafish. (B) Spastin protein truncation resulting from CRISPR/Cas9 in KO model. (C) Dechorionation efficiency rate in 60 hpf embryos wild-type (Ctrl) compared with Spastin KO (Spa-/-). N = 3 experiments; n > 200 embryos per group. (D-E) Impact of Spastin deletion on oxygen consummation rate (OCR) and extra-cellular acidification rate (ECAR) in 48 hpf embryos. n = 11 embryos per group. (F-H) Length, weight, and Body Mass Index in 3 months old adult zebrafish. n = 7 animals per group. Bars are mean ± SEM, ^#^*P =* 0.07, *P < 0.05, **P < 0.01, ***P<0.001 (unpaired *t*‐test).

Because of the localization of some isoforms to LDs, we next investigated the impact of Spastin on metabolic properties by designing a knockout (KO) zebrafish line based on CRISPR/Cas9 strategy. The gRNA targeted the second exon, immediately after the second initiator codon (M61), resulting in a truncation affecting the four main isoforms (Fig 2A and 2B). In embryos, the lack of Spastin did not affect development or morphology, but altered dechorionation, which was delayed in 30% of the animals at 60 hpf (Fig 2C). Respirometry, performed with the Seahorse XF24 Extracellular Flux Analyzer, revealed a reduction of basal oxygen consumption in 48 hpf KO embryos and a similar tendency in maximal respiration (Fig 2D), indicating reduced mitochondrial respiration. The extracellular acidification rate was also decreased in Spastin depleted embryos compared with wild-type fish (Fig 2E), suggesting a reduced reliance on glycolysis [50]. In adults, lack of Spastin reduced size, weight and BMI in 3 months old KO fish compared with wild-type littermates (Fig 2F–2H). These results highlight the metabolic function of Spastin.

### Spastin depletion affects LDs and ER patterning

**Fig 3:**
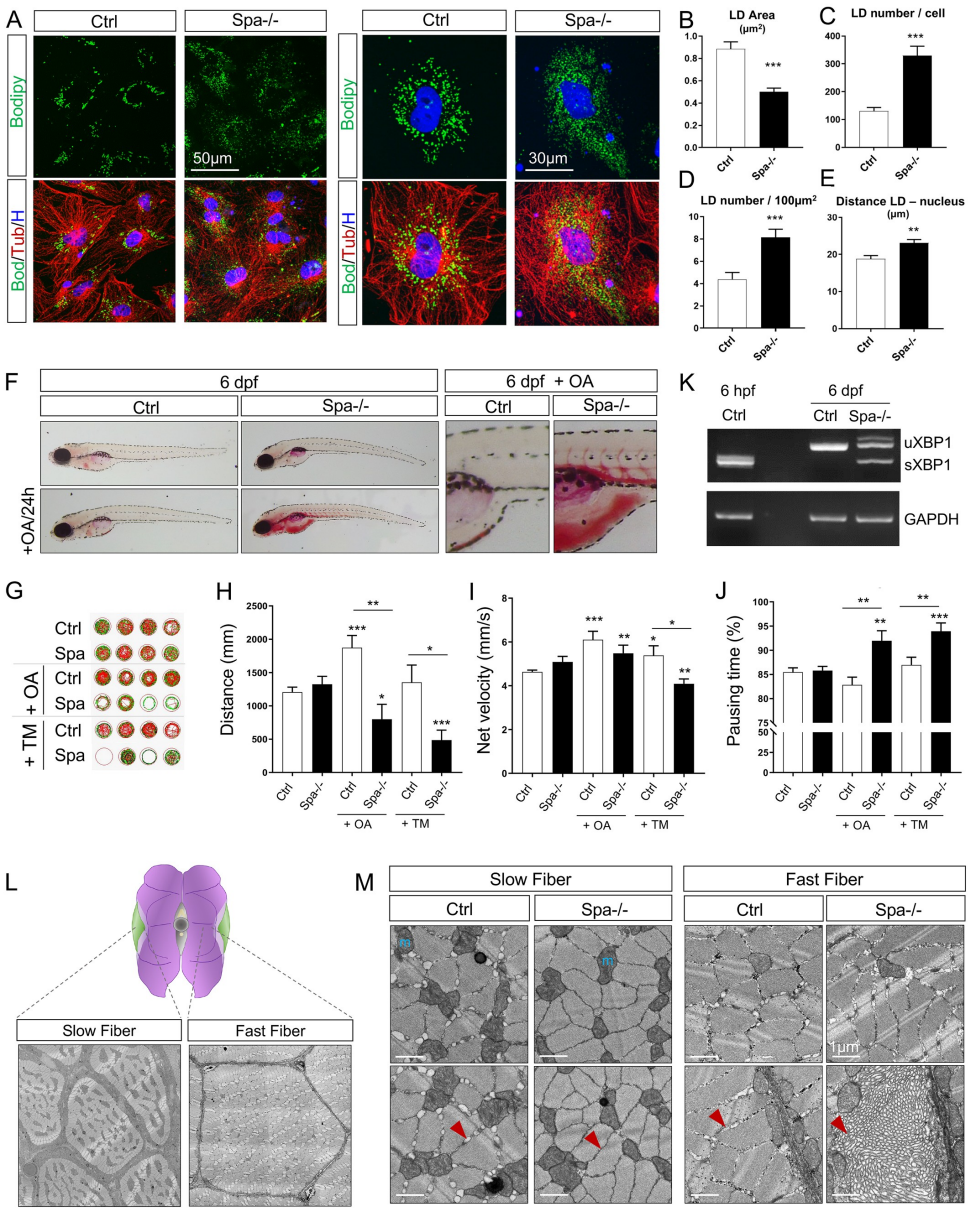
Spastin knockout affects lipid droplets and reticulum dynamics. (A) Confocal microscopy images of zebrafish embryonic cells from wild-type and Spastin KO animals (Ctrl and Spa-/-) treated with 300μM oleic acid (OA) for 18h before acquisition. Tubulin labeling corresponds to microtubules (red), Bodipy to lipid droplets (green) and Hoechst to nucleus (blue). (B) Quantification of LD area in embryonic cells. n = 23 cells Ctrl and n = 31 cells Spa-/-. (C-D) Quantification of LD number per cell (C) or in 100μm^2^ (D). n = 23 cells Ctrl and n = 31 cells Spa-/-. (E) Quantification of mean distance between individual LD and nucleus center. n = 23 cells Ctrl and n = 31 cells Spa-/-. (F) Oil red O staining in 6 dpf zebrafish larvae. Wild-type and Spastin KO were compared in basal conditions or after a 24h of OA in fish water. N = 3 experiments; n = 10 embryos per group. (G-J) Locomotion analyses in 6 dpf larvae (n = 24 larvae in each group) and after 24h administration of OA or Tunicamycin (TM) in fish water (n = 12 in each group). Zebrabox quantifications based on motion detection thresholds distinguishing slow (v) and fast velocity (V) as follow: 0<v<3mm/s (green) and V<6m/s (red). (K) RT-PCR detection of XBP1 splicing in 6 dpf zebrafish. (L) Schematic representation of slow versus fast twitch skeletal muscle distribution in adult zebrafish with respective electron micrographs delineating one fiber. (M) Electron micrographs taken in 8 months zebrafish skeletal muscle. Red arrow: sarcoplasmic reticulum, m: mitochondria. Bars are mean ± SEM, *P < 0.05, **P < 0.01, ***P<0.001 (unpaired *t*‐test vs. Ctrl or as indicated with by an horizontal line).

Given the impact of Spastin on metabolism and the potentially important role of M1 isoforms targeting LDs [30], we continued with the exploration of subcellular components. Here, we incubated embryonic cells from 24 hpf zebrafish embryos for 18h with 300μM of oleic acid (OA) to stimulate the production of LDs. Immunolabeling of Tubulin did not show any differences in MT structure in KO conditions compared with control. BODIPY staining pinpointed dramatic modifications of the LD network in Spa-/- cells (Fig 3A). Lack of Spastin decreased LD size by two-folds and increased their number per cell from 131±13 to 330±34 (Fig 3B and 3C). This increase was confirmed by measuring LD density in 100μm^2^ (Fig 3D). Further, the dispersion from the nucleus center was significantly higher with a mean distance between LD and nucleus of 18.8±0.9μm in wild-type cells and 23.1±0.9μm in KO cells (Fig 3E). In summary, Spastin depletion induced a higher number of LDs, with smaller caliber, that were more disseminated and dispersed through the cytoplasm.

To decipher the impact of LD reorganization *in vivo*, we followed fat distribution in 6 dpf larvae with Oil Red O staining [51]. At this developmental stage, neither adipose depots nor guts are yet developed. In basal conditions, Spastin KO larvae exhibited lipid accumulation around the swim bladder compared with control animals (Fig 3F). Administration of OA [52] from 5 to 6 dpf triggered an accumulation of lipids in the head, around the yolk and in the vessels (Fig 3F) in Spastin KO fish, while control larvae succeeded in buffering the accumulation of fat (Fig 3F).

To determine functional consequences, we measured locomotor performances in 6 dpf larvae (Fig 3G–3J). In basal conditions, wild-type and Spa-/- fish exhibited similar swimming capacities. While OA administration increased the distance swam by control fish from 1206±76 to 1873±184mm in 30min, Spastin KO larvae presented a significant reduction from 1324±119 to 799±224mm (Fig 3H). OA treatment also reduced net velocity and increased pausing in Spa-/- animals (Fig 3I and 3J). Given that the impact of OA on Spastin mutant could be partially mediated by ER stress [52, 53], we evaluated the expression of X-box Binding Protein 1 (XBP1) by RT-PCR [54]. Indeed, the presence of spliced XBP1 (sXBP1), in addition to the canonical form (uXBP1), is an indicator of ER stress as shown in 6 hpf embryos [54], here used as positive controls (Fig 3K). In Spastin KO larvae, the detection of sXBP1 indicated the activation of ER stress response (Fig 3K), which was not present in wild-type controls. We further confirmed the higher sensitivity to ER stress of Spastin KO treating the larvae for 24h with 2μg/ml of Tunicamycin (TM). TM administration did not affect the distance swam in control larvae but impacted Spastin KO which exhibited a large reduction of mean distance and net velocity (Fig 3G and 3I) with a reciprocal higher pausing time (Fig 3J). This confirms that Spastin KO larvae are more sensitive to ER stress than controls.

This impact of Spastin on lipid management and ER stress at the larval stage called for the investigation of organelles in adult fish with a particular interest in skeletal muscle. Indeed, the role of Spastin in non-neuronal tissues remains poorly characterized, despite its ubiquitous pattern of expression (Fig 1C) and the previous description of Spastin effect on human skeletal muscle transcriptome [55]. We used electron microscopy to decipher the role of Spastin on zebrafish skeletal muscle ultrastructure (Fig 3L). In control animals, slow muscle was enriched in mitochondria around fibers and between sarcomeres (*i*.*e*. the contractile units), while fast muscle mitochondria were mostly at the periphery (Fig 3L). Lack of Spastin did not affect mitochondria appearance (Fig 3M). In wild-type slow and fast muscle, sarcomeres were surrounded by budding ER (Fig 3M). Lack of Spastin completely flattened ER in slow fibers and accumulated ER in hives at the edges of fast fibers (Fig 3M). These observations confirmed the importance of Spastin in muscle and in organelle organization. Taken together, these experiments, performed in the zebrafish model, confirmed the important impact of Spastin on LDs and ER in different tissues and cell types.

### Human Spastin M1 isoforms conserve features of the zebrafish counterparts

**Fig 4:**
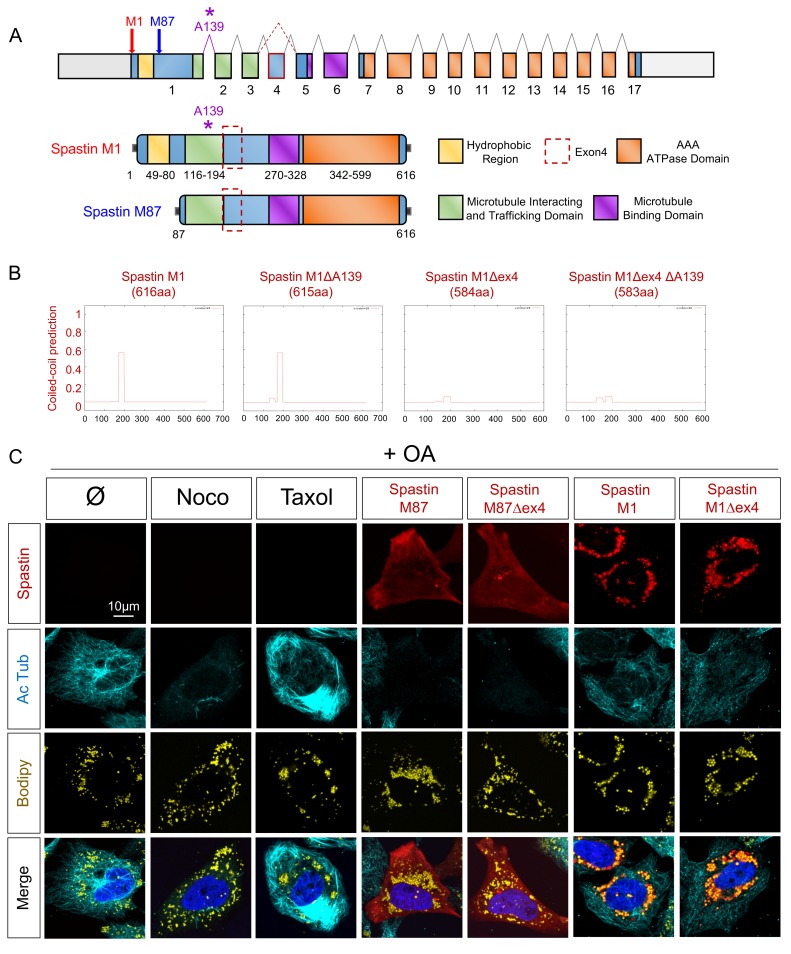
Spastin isoforms in human. (A) Schematic representation of human Spastin splice variants. (B) Coiled-coil prediction in Spastin isoforms (designed from https://embnet.vital-it.ch/software/COILS_form.html). X-axis corresponds to the amino-acids sequence. (C) Confocal microscopy images of HeLa cells overexpressing Spastin splice variants treated with oleic acid (OA) for 18h. Nocodazole (Noco) and Taxol administration (12h) serve as control conditions. Cherry-tagged Spastin appears in red. Acetylated Tubulin labeling corresponds to microtubules (cyan), Bodipy to LDs (yellow) and Hoechst to nucleus (blue). See also S2 Fig.

To emphasize the observations obtained in zebrafish, the impact of human Spastin isoforms on organelles had to be investigated. Analysis of cDNA from human muscle confirmed the coexistence of the two initiator codons and the splice modifications that suppressed exon 4 as described in zebrafish (Fig 4A). The absence of exon 4 was systematically accompanied by the lack of Alanine 139 (A139), which is encoded by the overlapping sequence formed by exon 1 and 2 (Fig 4A). The lack of exon 4 abolished the prediction of a strong coiled-coil domain in human Spastin (Fig 4B). The removal of A139 residue had a minor impact on this secondary structure (Fig 4B). Given these observations, we pursued the investigations on the full-length isoforms M1 and M87, as well as their counterpart missing both exon4 and A139 (hereafter referred as to M1Δex4 and M87Δex4).

In HeLa cells treated with 300μM of OA, M87 and M87Δex4 exhibited a diffuse location and triggered the complete severing of acetylated Tubulin in all the transfected cells, comparable to a 12h treatment with nocodazole. M1 and M1Δex4 presented an exclusive targeting to LDs and partially conserved the severing activity (Fig 4C). These results pointed out strong and evolutionary-conserved functions of the different isoforms of Spastin.

To decipher the putative involvement of the long form M1 and M1Δex4 in HSP, we reproduced the mutation C488Y in our constructs, as well as a new modification that we identified in human cDNA H455R. In HeLa cells, both mutations converged in strong effects targeting both LDs and MTs, similarly for M1 and M1Δex4 (S2A Fig). LDs were accumulated around the nucleus. In presence of mutant constructs, acetylated Tubulin formed large concentric bundles. Mutant Spastin presented a specific pattern at the cortical extremity of MTs, forming an inverse gradient of intensities with acetylated Tubulin (S2A Fig), *i*.*e*. when one fluorophore was intense the other was moderate and vice-versa.

In challenged conditions, mutant M1^H455R^ was accumulated on MTs around the nucleus in presence of Taxol. Nocodazole led to a strict relocation of Spastin on LDs. M1^H455R^ maintained MTs-targeting despite a short cold exposure that suppressed the acetylated Tubulin signal [56] (S2B Fig). In summary, mutations in Spastin long isoforms converged on a common gain of function that induced bundled MTs, equivalent to an abnormal stabilization, and modifications of the LD network.

### Spastin isoforms modulate organelles contact sites

**Fig 5:**
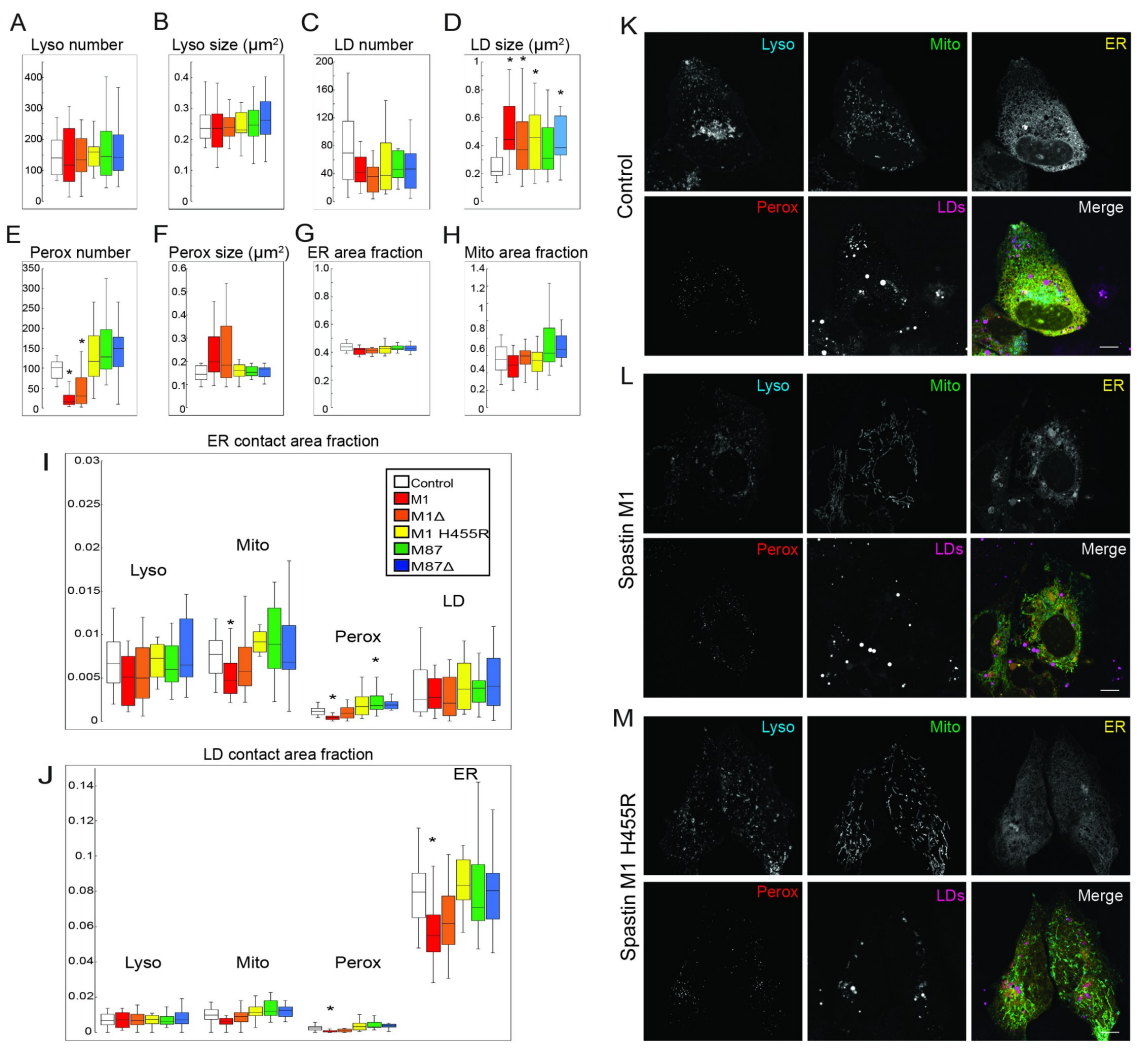
Wild-type and mutant Spastin affect organelle networks. Multispectral analysis of organelle size and number in U2OS cells expressing humans Spastin isoforms M1, M1Δex4, M87, M87Δex4 and the mutant M1^H455R^. (A-B) Quantification of lysosome (Lyso) number per cell and median size. (C-D) Quantification of LD number per cell and median size. (E-F) Quantification of peroxisome (Perox) number per cell and median size. (G) Quantification of ER area per cell. (H) Quantification of mitochondria (Mito) area per cell. (I) Quantification of organelle contact area fraction centered on ER. (J) Quantification of organelle contact area fraction centered on LDs. (K-M) Representative images of U2OS cells expressing mock (K), human Spastin M1 (L) and the mutant counterpart M1^H455R^ (M). Scale bars, 10 μm. Box whisker plots, statistical tests are unpaired *t*-tests with Bonferroni correction.

To explore the fate of other organelles with a quantitative approach, we used multispectral analysis in transfected U2OS cells [20] (Fig 5A–5M). Overexpression of Spastin isoforms increased LD size (Fig 5C and 5D). M1 and M1Δex4 decreased peroxisome number (Fig 5E) but did not modify their size (Fig 5F). Lysosome number and size were not affected (Fig 5A and 5B). ER and mitochondria area fraction remained stable (Fig 5G and 5H) even when ER morphology was altered (Fig 5K–5M).

In addition to these modifications of individual organelles, Spastin isoforms modulated contact sites. Wild-type M1 reduced the contact between ER/mitochondria and ER/peroxisome (Fig 5I). Similarly, M1 decreased contact between LD/peroxisome and LD/ER (Fig 5J). In the absence of exon 4 or the presence of the mutation H455R, the impact of Spastin M1 on organelle contacts was not observed (Fig 5I and 5J).

**Fig 6:**
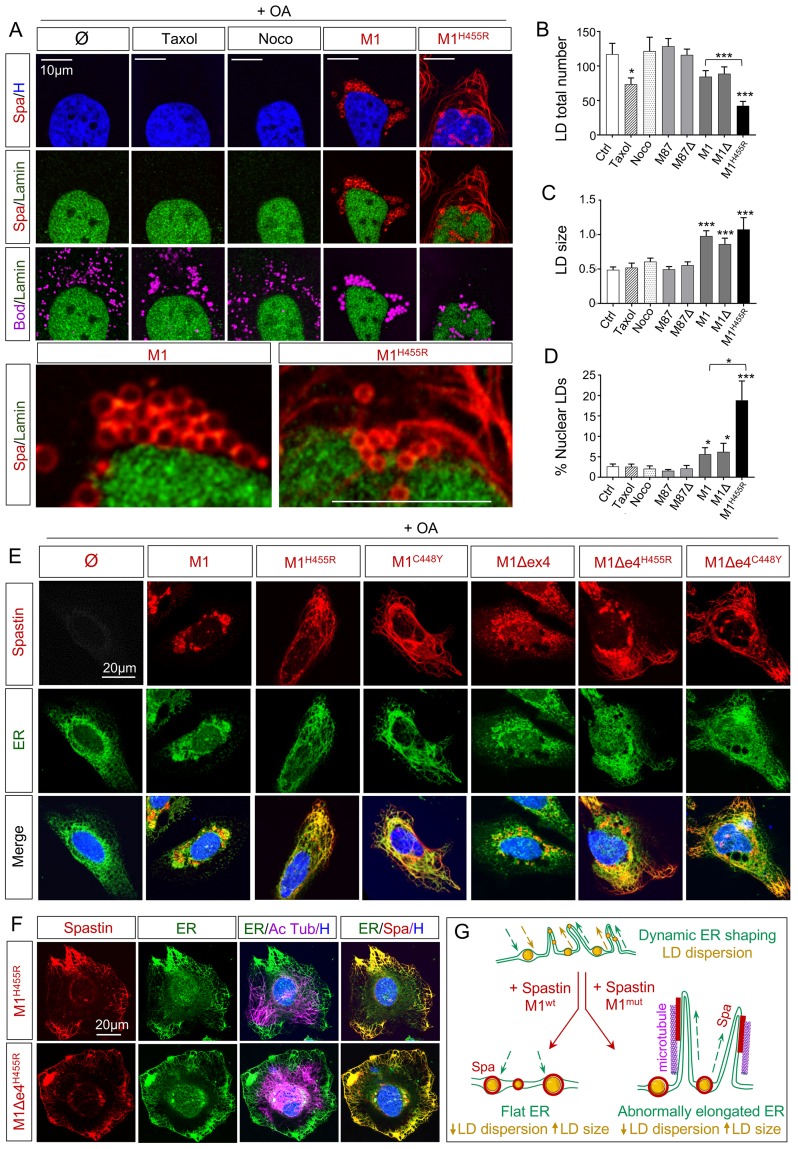
Spastin isoforms modulate LDs and ER shape. (A) Confocal microscopy images of HeLa cells overexpressing human Spastin M1 wild-type and mutant (H455R) treated with oleic acid (OA) for 18h. Nocodazole (Noco) and Taxol administration (12h) serve as control conditions. Cherry-tagged Spastin appears in red. Lamin A/C labeling corresponds to the nuclear compartment (green), Bodipy to LDs (magenta) and Hoechst to nucleus (blue). N = 4. (B-C) Quantification of LD number and size in HeLa cells treated with OA. n = 10 to 15 cells per condition. (D) Quantification of nuclear LDs in HeLa cells treated with OA. n = 10 to 15 cells per condition. (E-F) Confocal microscopy images of HeLa cells overexpressing human Spastin M1 isoforms treated with OA for 18h. Cherry-tagged Spastin appears in red. Calcineurin labeling corresponds to ER (green), acetylated Tubulin to microtubules (magenta) and Hoechst to nucleus (blue). (G) Schematic representation of ER shaping in presence of wild-type and mutant Spastin with subsequent impact on LD dispersion. In basal conditions, ER (green) follows a dynamic balance of elongation/retraction controlling the distribution of associated LDs (yellow) through the cell. Overexpression of wild-type Spastin M1 (red) triggers the retraction of ER leading to the accumulation of bigger LDs close to the nucleus. Mutated Spastin M1 (red) induces an abnormal elongation of ER along microtubules (magenta), resulting in a reduction of LD dispersion. Bars are mean ± SEM, *P < 0.05, **P < 0.01, ***P<0.001 (unpaired *t*‐test vs. Ctrl or as indicated by a bracket).

To complete this approach, we used a quantitative analysis in HeLa cells treated with OA to enhance LD biogenesis. Expression of wild-type M1 and M1Δex4 increased LD size (Fig 6A–6C). In a pathological context, the presence of H455R mutation decreased LD number and increased LD size. These observations were coherent with the effect of Spastin KO described in zebrafish cells (Fig 3A–3E). M87 and M87Δex4 did not impact LDs features, similarly to the administration of nocodazole, suggesting that the effect of Spastin could not be attributed to the severing activity on MTs (Fig 6A–6C). Stabilization of MTs by Taxol administration reduced LD number without changing their size, confirming that Spastin effect was not limited to Tubulin modulations (Fig 6A–6C).

In addition to the modification of LD size and number, Spastin long isoforms triggered a redistribution of LDs that accumulated around the nucleus (Fig 6D). High magnification confirmed a close contact between Spastin rings and Lamin (Fig 6A). Expression of M1 and M1Δex4 increased the contact between LDs and nucleus membrane, but also enhanced the accumulation of intranuclear LDs that represented respectively 5.6±1.6 and 6.2±2.1% of the total pool compared to 2.6±0.6% in control conditions (Fig 6D). Mutations of Spastin M1 massively increased intranuclear LDs to 18.8±4.7%. The impact of Spastin on nuclear LDs was in line with a former study reporting an effect of the protein on nuclear membrane [57].

### Spastin impacts LDs through effects on ER

The impact of Spastin on the LD network cannot be explained by MTs modulation but could be due to an alteration of biogenesis mechanisms. It is now well established that nascent LDs appear through neutral lipid accumulation within the ER in a complex budding process mediated by key players such as Seipin [11, 12]. The modulation of this machinery is still poorly characterized. To follow up on the multispectral analysis pointing to a decreased contact between ER/LD (Fig 5J), we performed a complementary staining of Spastin, Calreticulin and acetylated Tubulin in HeLa cells (Fig 6E and 6F). While wild-type Spastin M1 and M1Δex4 resulted in a retraction of ER around the nucleus, mutant counterparts broadly reorganized the network in filamentous structures (Fig 6E). Co-staining of acetylated Tubulin confirmed that Calcineurin labeling colocalized with mutated Spastin along the cortical extremity of MTs (Fig 6F). Our observations are in line with previous works showing that Spastin M1 isoform can modulate ER shaping through the anchoring of its hydrophobic N-terminus [58, 59].

Here, we propose that the retraction or elongation of ER along MTs determine the position of LD biogenesis in the cell (Fig 6G). As the majority of LDs remain attached to the ER [20], the impact of Spastin on ER spreading may directly define LD dispersion. In wild-type context, long Spastin isoforms modulated ER shaping by its retraction around the nucleus, explaining the accumulation of fewer but bigger LDs, further participating in the presence of perinuclear LDs. Mutations spread ER along MTs to form excessive tubular shapes, resulting in the same LDs pattern and size than wild-type Spastin (Fig 6G).

### Spastin modulates other players involved in HSP at both transcript and protein levels

**Fig 7:**
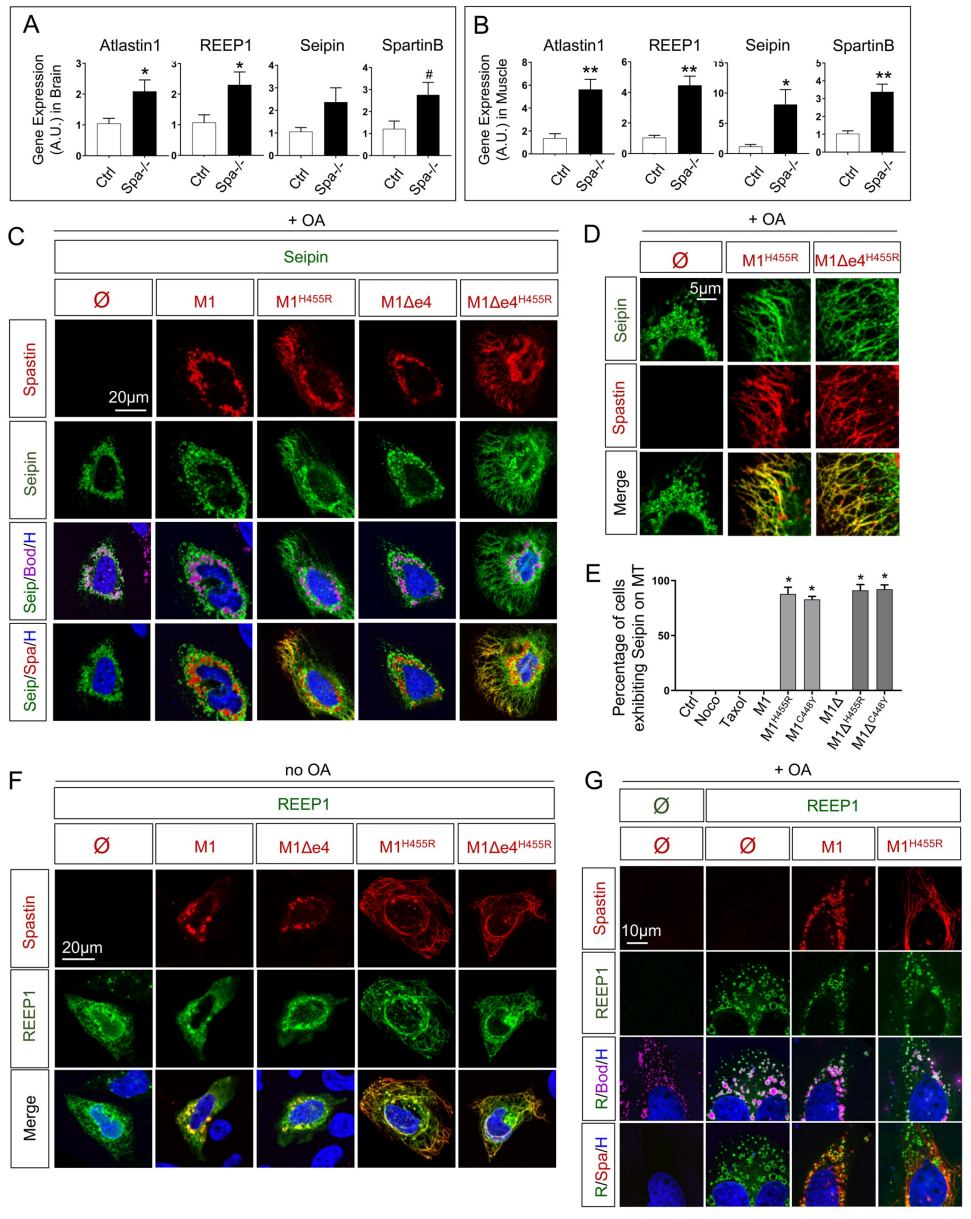
Spastin influences transcription and location of HSP-related proteins to control ER/LD dynamics. (A) Quantification of REEP1, Atlastin1, Spartin and Seipin transcript by qPCR in 8 months old zebrafish brain. n = 4 animals per group. (B) Quantification of REEP1, Atlastin1, Spartin and Seipin transcript by qPCR in 8 months old zebrafish skeletal muscle. n = 4 animals per group. (C-D) Confocal microscopy images of HeLa cells overexpressing human Seipin with Spastin M1 isoforms (wild-type and mutants) treated with oleic acid (OA) for 18h. Cherry-tagged Spastin appears in red, Seipin in green, LDs (Bodipy) in magenta and nucleus (Hoechst) in blue. (E) Percentage of cells exhibiting tubular ER along MTs. (F-G) Confocal microscopy images of HeLa cells overexpressing human REEP1 with Spastin M1 isoforms in basal conditions (F) or after 18h administration of OA (G). Cherry-tagged Spastin appears in red, REEP1 in green, LDs (Bodipy) in magenta and nucleus (Hoechst) in blue. Bars are mean ± SEM, ^#^*P* = 0.08, *P < 0.05, **P < 0.01, ***P<0.001 (unpaired *t*‐test). See also S3 Fig and S4 Fig.

ER shaping is known to be modulated by other proteins involved in HSP such as Atlastin1 and REEP1 [60–63]. Our results reinforce the hypothesis of a global function shared by HSP-associated proteins to coordinate ER shaping and MTs dynamics [64]. To determine the potential connection in a common pathway, we investigated the impact of Spastin depletion on other genes transcripts. We chose to focus on four HSP-associated proteins: Seipin (OMIM# 606158, involved in SPG17 OMIM# 270658) and Spartin (OMIM# 607111, involved in SPG20 OMIM# 275900) given their role in LD fate [65], as well as Atlastin1 (OMIM# 606439, involved in SPG3A OMIM# 182600) and REEP1 (OMIM# 609139, involved in SPG31 OMIM# 610250) given that both had been shown to impact LD size [64, 66, 67]. Thus, we followed the expression of Seipin, Spartin, Atlastin1 and REEP1 (also referred as to SPG17, SPG20, SPG3A and SPG31 respectively) in Spastin KO zebrafish (Fig 7A and 7B). In brain, REEP1 and Atlastin1 presented a twofold increase in absence of Spastin. Seipin and Spartin exhibited a similar trend (Fig 7A). In skeletal muscle, Spastin depletion induced a threefold increase of Spartin and Atlastin1. Seipin and REEP1 transcripts appeared respectively 7 and 4 times more present in Spa-/- than in controls (Fig 7B). These results point to a transcriptional compensation of Seipin, Spartin, Atlastin1 and REEP1 to face Spastin depletion, thus providing evidence for a genetic link between Spastin and other HSP-related genes.

In addition to the transcriptomic dialogue between HSP genes, we examined the influence of Spastin on these candidates at the protein level defining their localization in HeLa cells. Wild-type Spastin M1 and M1Δex4 modulated Seipin through ER shaping, decreasing its location around LDs. Conversely, mutated Spastin proteins spread Seipin along MTs to the cell periphery (Fig 7C and 7D). This effect was specific to Spastin mutations, as MT-modifying drugs were unable to relocate Seipin (Fig 7E). The impact of Spastin was also observable for a longer splice variant of Seipin [11] (S3A and S3B Fig). The relocation of Seipin in presence of wild-type or mutant Spastin confirmed that the reticulum reorganization affects the machinery responsible of LD biogenesis, explaining the dispersion or the retraction around the nucleus.

Spastin also affected other key players of the ER/LD dialogue. Expression of wild-type Spastin M1 or M1Δex4 triggered the relocation of REEP1 (Fig 7F) depending of the presence or absence of OA. In the absence of OA, REEP1 relocated along the ER, thus around the nucleus (Fig 7F). Expression of mutant Spastin M1^H455R^ and M1Δex4^H455R^ redistributed REEP1 along thick tubules of ER (Fig 7F). Remarkably, in HeLa cells, REEP1 property to shape tubular ER was very weak, especially compared with mutant Spastin. OA administration totally relocated the protein around giant LDs close to the nucleus (Fig 7G) revealing an unexpected LD-targeting of REEP1, which also enhanced the volume of these organelles (S3C Fig). This property was not dependent on Spastin, as REEP1 still targeted LDs in zebrafish embryonic cell depleted for Spastin (S3D Fig). In presence of OA, wild-type Spastin M1 and REEP1 colocalized on LD surface, with REEP1 exhibiting an exclusive pattern at the inter-LD contact sites. Mutated Spastin M1^H455R^ (Fig 7G) was excluded from the LD surface in presence of REEP1 (Fig 7G).

Wild-type and mutant Spastin M1 relocated Atlastin1 as a function of ER shaping (S4A Fig). Spartin expression generated clusters of LDs in which wild-type and mutant Spastin M1 were localized (S4B Fig). Altogether these elements point to a strong interdependence between HSP-associated proteins. However, Spastin represents the core of this machinery and plays a crucial role in ER shaping and subsequent LD location.

### Spastin depletion triggers alterations in the lipid profile

**Fig 8:**
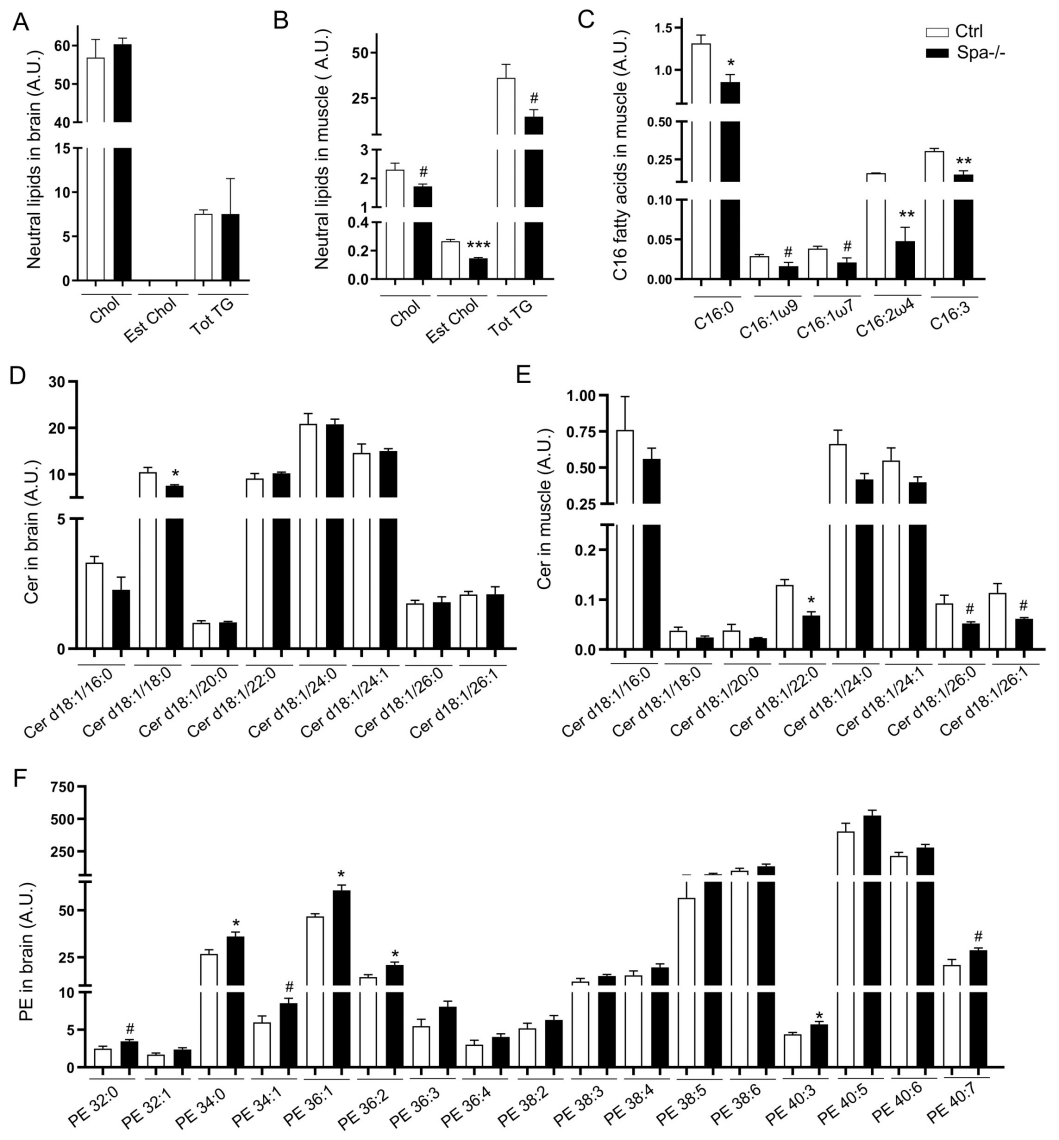
Spastin depletion modifies lipid profile in adult zebrafish brain and skeletal muscle. (A-B) Unesterified cholesterol (Chol), total esterified cholesterol (Est Chol) and total triacylglycerides (Tot TG) in brain (A) and in muscle (B). (C) Fatty acids composed of C16 in muscle. (D-E) Ceramides (Cer) in brain (D) and muscle (E). (F) Phosphatidylethanoamines (PE) in brain. All values correspond to lipid quantity per total protein amount. Bars are mean ± SEM (n = 3 per group), #*P*<0.08, *P<0.05, *P*<0.01, ***P<0.001 (unpaired *t*‐test between Ctrl and Spa-/-). See also S1 Table.

The effect of Spastin on LDs and ER suggested a potential impact on neutral lipids or membrane components. Using state-of-the-art mass spectrometry, we analyzed lipidomics in brain and skeletal muscle from wild-type or Spastin KO zebrafish. Quantification of non-esterified cholesterols (Chol), esterified cholesterols (Est Chol) and triacylglycerides (TG) did not reveal any alterations of neutral lipids in Spa-/- brain (Fig 8A and S1 Table). In skeletal muscle, Spastin KO induced reductions of Chol, Est Chol and total TG (Fig 8B and S1 Table). Analysis of saturated, mono- and poly-unsaturated fatty acids (respectively SAFA, MUFA and PUFA) did not indicate any variations between control and Spa-/- brain but were affected in skeletal muscle (S1 Table). Among the different molecular species, only fatty acids composed of 16 carbons were reduced in the absence of Spastin (Fig 8C). Altogether, these results highlighted the impact of Spastin on neutral lipids and associated fatty acids in skeletal muscle. The broad reduction of TG and Est Chol may be associated with LDs alteration and may represent a new hallmark of Spastin depletion.

Due to the involvement of Spastin in organelle dynamics and contacts, we also investigated its impact on structural lipids. Analysis of phospholipids demonstrated that phosphatidylcholines (PC) were not affected in Spastin KO fish (S1 Table). Sphingomyelins (SM) and Phosphatidylinositols (PI) exhibited few modifications in some specific species. SM d18:1/16:0 presented a twofold reduction in Spastin KO skeletal muscle, while PI 38:2 increased in KO brain (S1 Table). Beside these specific modifications, Ceramides (Cer) were broadly affected by Spastin depletion with a reduction of Cer d18:1/18:0 in brain (Fig 8D) and Cer d18:1/22:0, Cer d18:1/26:0 and Cer d18:1/26:1 in muscle (Fig 8E). Phosphatidylethanolamines (PE) also revealed dramatic alterations in Spastin KO fish, specifically in the nervous system. PE 32:0, PE 34:0, PE 34:1, PE 36:1, PE 36:2, PE 40:3 and PE 40:7 followed a comparable increase in Spa-/- brain (Fig 8F and S1 Table). Altogether, these finding confirmed the impact of Spastin on structural lipids with organ specificities. Interestingly, Spastin affected particularly PE and Cer, two major components of organelle membranes known to impact membrane fluidity and folding, notably in the ER [68, 69].

## Discussion

### Spastin isoforms coordinate cytoskeleton and organelles

While the role and location of a protein are historically characterized from its canonical isoform, recent advances reveal that alternative splice variants can represent a source of function diversity [70]. In pathological conditions, splice isoforms are associated to specific disorders as described for Tau [71]. The co-existence of different Spastin variants has been described from worm to human [43]. In human, former studies mainly focused on the short M87 protein, known to carry the severing property [32, 33, 72]. However, recent advances highlighted that the M1 long isoform harbors a LD targeting sequence [30]. In addition to the existence of the two start codons, an alternative splicing of exon 4 is also conserved from fish [48] (Fig 1C–1E) suggesting a specific function for this variation. The presence of exon 4 represents a strong coiled-coil domain (Fig 4B) and has been hypothesized to modify Spastin functions [73]. Indeed, while M1 and M1Δex4 share similar effect on peroxisome number and LDs size (Fig 5C–5F and 6B–6D), M1 exhibits a unique propensity to reduce organelle contact area (Fig 5I and 5J).

Beyond these major isoforms, the existence of uncharacterized splice events present in low concentration may confer new levels of complexity. In zebrafish, a new splice variant of M1, deleted from exon 2 to 10, only conserves LD targeting properties (S1 Fig) and may play a role of competitor for the full-length M1. Further investigations should confirm the existence of this variant in mammals. Conversely, low abundance isoforms lacking exon 8 and 15 have been described in human, but their functions remain unknown [74]. The alteration of AAA domains may confer to these natural variants a MT-bundling function similar to mutant Spastin. These results highlight the complexity of Spastin variants.

### Spastin plays an important role in muscle

While different papers claimed a specific expression of Spastin, notably M1 isoform, in the central nervous system [75], we provide evidence of the ubiquitous pattern of this protein. Spastin depletion affects respiration, locomotion and body measurements. At the cellular level, KO fish reveal an impairment of sarcoplasmic reticulum (Fig 3M) and confirm an impact of Spastin on skeletal muscle [55]. Lipid profile analyses highlight a reduction of neutral lipids in muscle, while the nervous system is not affected. C16 fatty acids and associated TG are specifically altered in skeletal muscle and may represent a new biomarker in HSP patients. Altogether these observations indicate the involvement of Spastin in muscle. The action of Spastin and other HSP genes on organelles may affect tissue metabolism and participate in the muscular symptoms described in patients [35].

### Spastin coordinates MTs and ER shaping to modulate LDs dispersion

Spastin isoforms reveal a global impact on organelles (Figs 5 and 6) that cannot be limited to their action on MTs dynamics. Spastin depletion alters ER morphology in fish skeletal muscle while overexpression of M1 and M1Δex4 condensate ER around the nucleus in mammalian cells. A direct role of Spastin on ER shaping has been recently evidenced [58, 59]. Here we confirm this function *in vivo* (Fig 3M) and implicate Spastin deletion in causing ER stress (Fig 3F–3K).

The reorganization of the ER network affects contacts and dynamics of other organelles, mainly LDs. In KO zebrafish cells, lack of Spastin enhances the dispersion of small LDs (Fig 3A and 3B), while overexpression of M1 isoforms in HeLa cells promote LD accumulation around the nucleus (Figs 4C, S2A and 6A–6D). Spastin appears as an inhibitor of LDs dispersion. Although MTs have been shown to impact LD location in the cell [27], Taxol and nocodazole administration do not mimic Spastin effects (Fig 6B–6D) confirming that the impact of the protein on LD dispersion is not mediated by its action on cytoskeleton. As the large majority of LDs remain in contact with ER after biogenesis [20], the reorganization of reticulum along MTs network subsequently determines the dispersion of LD. We here propose the concept that by controlling the spreading of ER, Spastin confers to LDs a specific trafficking process, different from other organelles that move directly along MTs tracks. LDs spread in the cell in function of ER/MTs dynamics (Fig 6G). Given that the elongation of ER along MTs represents an evolutionary conserved mechanism that has been described in plants [76], the subsequent spreading of LDs is probably an ancestral process that should be further investigated in eukaryotes. In mammals, this dispersion may be crucial to deliver LDs to the extremity of long neurons such as upper motor neurons. However, LDs also play key functions in other cell types [1, 2, 5, 9, 77] explaining the impact of Spastin on many tissues.

Structurally, modulations of ER shape and LD dynamics result in organ-specific alterations of the lipid profile. Spastin impacts not only LD number but also affects their composition (Fig 8). Structural lipids are also modified, notably Cer and PE. These phospholipids, which are particularly important to define membrane fluidity, may participate in ER shaping and organelle contact. PEs are also chaperone lipids that ensure protein folding and participate in ER stress [68, 69]. These findings show that Spastin functions are linked with the modulation of the lipid profile in neuronal and muscle tissues.

### Mutations in Spastin massively affect organelles through M1 isoforms gain of function

HSP-related mutations were reported in the different domains of Spastin [34]. These mutations lead to the loss of M87 function, which represented a discrepancy with the dominant transmission of the disease, did not affected only the AAA domain and did not explain HSP pathological features [78]. Other studies highlight the importance of M1 isoforms in the disease onset [40] with mutations conferring to this isoform a bundling activity that stabilizes MTs [79]. Here, we reinforce the hypothesis that long Spastin isoforms exhibit a gain of function and may play a central part in HSP physiopathology [78, 79]. In mutant conditions, M1 and M1Δex4 converge on an abnormal stabilization of MTs (S2 Fig). This propensity to bundle MTs may co-exist with the severing activity in the wild-type protein [73], but becomes dominant in mutants.

Spastin M1 gain of function is not only associated with cytoskeleton stability. Mutations in Spastin long isoforms also impacted LD dispersion and morphology (Fig 6A–6D). The abnormal formation of tubular ER [59] (Fig 6E–6G) and the relocation of other proteins involved in LD biogenesis (Fig 7D) resulted in the reorganization of organelle networks, notably LDs. Surprisingly, excessive ER spreading and retraction converged on similar consequences for LDs.

Mutated M1 also increased the accumulation of LD around the nucleus (Fig 6A and 6D). The alteration of ER shaping may enhance the import of LDs inside the nucleus or promote biogenesis directly inside the nuclear membrane. Former studies highlighted an action of Spastin on nuclear envelope integrity [57] that may participate in LD accumulation in the nucleus. Regarding the numerous functions of LDs in the nucleus [80], it could be hypothesized that their abnormal accumulation may play a role in HSP pathophysiology.

### Spastin is involved in a multi-protein system controlling organelle fate

Based on multiple domains and isoforms, Spastin coordinates MTs, ER and LDs in basal and pathological conditions. Strikingly, proteins sharing similar functions are all involved in the same group of diseases. Spastin influences HSP-related proteins at the transcriptomic level (Fig 7A and 7B) and determines their relocation in the cell (Fig 7C–7G).

REEP1 [62–64] and Atlastin1 [61, 81] are known to influence ER shape by creating tubular structures, similarly to mutant Spastin M1. By controlling the location and the transcription of these HSP-related proteins (Figs 7 and S4A), Spastin coordinates ER shape. The convergent functions between Spastin and REEP members are probably crucial for neuronal and muscle tissues. In cardiac muscle from rat, inactivation of REEP5 (OMIM# 125265) induces an alteration of sarcoplasmic reticulum morphology [82], comparable to the effect of Spastin deletion in fish muscle (Fig 3M). Among other Spastin partners, Seipin may represent the main actor to control LD dynamics. Transcriptional modulation of Seipin (Fig 7A and 7B) and relocation in function of ER state (Figs 7C–7E and S3A and S3B) affect LD biogenesis and subsequent dispersion through the cell.

Spastin uses MT tracks to remodel the ER and this shaping defines LD dispersion. While the impact of Spastin on MTs is well described, other HSP-related proteins also revealed an action on cytoskeleton. In drosophila, Atlastin and Spartin have been shown to modulate MTs stability [83]. Similarly to Spastin, Kinesins KIF1A (OMIM# 601255), another protein involved in HSP [84], accumulates on specific MTs regions when mutated in its ATPase domain [85]. Interestingly, Kinesins have been shown to modulate reticulum spreading along MTs [86, 87]. Their effect on LD transport notably described in drosophila may be due to an indirect action on ER shaping [26].

Beside its impact on ER shaping, Spastin controls HSP-related partners directly involved in LD dynamics. Spastin follows the clustering of LDs in presence of Spartin (S4B Fig), a protein initially described at the mitochondria level [88] but also reported as a LD modulator [65]. Here, we also describe a strong targeting of REEP1 to LDs, increasing their size (Figs 7G and S3C) in presence of fatty acid stimulation. While REEP1 depletion has been previously described to modulate LD size and to be associated with lipodystrophy in mouse model [89], this report provides evidence for the first time of a direct targeting of the wild-type protein to LDs. The access of REEP1 on LDs probably depends on fatty acids concentration, as other studies described the protein on LDs only in mutant context [66]. Wild-type and mutant Spastin triggers subtle modification in REEP1 location.

Further investigations are needed to explore the participation in this dialogue between ER and LDs of other HSP-related proteins known to impact ER shaping [45], such as RTN2 (OMIM# 603183, involved in SPG12 OMIM# 604805) [90], Protrudin (OMIM# 610243, involved in SPG33 OMIM# 610244) [91] or ARL16IP (OMIM# 607669, involved in SPG61 OMIM# 615685) [92]. Indeed, we can hypothesize that by modulating ER shape, these proteins may impact LD dispersion. Furthermore, KIF1A may also play a role in this machinery, potentially representing the engine that actively spreads ER along microtubules as observed in plant and mammalian cells, with a subsequent reorganization of the LD network [76, 86].

Altogether these HSP-related proteins constitute a scaffold ensuring membrane shaping and organelle dialogues. Their numerous functions are supported by splice-mediated complexity, as illustrated by Spastin or Paraplegin variants [93]. This machinery converges to control LD dynamics. At the crossroad of MTs, ER and LDs, Spastin appears as a key player of this machinery and illustrates the functional variability carried by splice isoforms.

### Conclusion

In summary, the characterization of Spastin isoforms reveals the complex functions of this multi-faceted protein. Coordinating MTs and ER dynamics, Spastin modulates the LD network and dispersion in the cell. These crucial functions are based on key partners similarly involved in the physiopathology of HSP. In the light of this complex machinery, HSP appears as the first group of diseases directly due to a disorganization of organelles dynamics. A major consequence is the modification of the lipid profile in absence of Spastin. These alterations exposed in the zebrafish model suggest that lipidomic analyses may supply new biomarkers for HSP patients and open new therapeutic targets.

## Materials and methods

### Ethics statement

This study protocol was approved by the cantonal veterinary authorities (approval number VD3391).

### Zebrafish Husbandry and strains

Zebrafish (*Danio rerio*, Oregon AB) were housed at the Zebrafish facility of the School of Biology and Medicine, maintained at 28.5°C and on a 14:10h light:dark cycle. Embryos were staged by hours (h) or days (d) post fertilization according to Kimmel *et al*. [94].

### Generation of CRISPR/Cas9 knockout

The guide RNA targeting zebrafish Spastin (Q6NW58) was designed on https://chopchop.cbu.uib.no. The target sequence was selected in exon 2 with a predicted efficiency of 0.74: TGTAGGTGATAAGCAGAAGGCGG. gRNA was produced and purified with Gene Art precision gRNA synthesis kit (A29377, Invitrogen, ThermoFischer Scientific, Waltham, MA, United States). gRNA and recombinant Cas9 nuclease (B25641, Invitrogen) were mixed at respectively 100ng/μl and 500pg/nl in a 200mM solution of KCl. 1nl of the mix was injected in the first cell of AB embryos.

F1 heterozygous animals were identified after fin clipping and PCR amplification of Spastin fragment using the following primer: For 5-ACCTGTGATGTGAAACGTTTCT-3; Rev 5-TTGTAATAAGCCGTGTAATAA-3. The sequence modification was visualized on Bis-acrylamide gels. The exact nature of the CRISPR/Cas9-induced mutation was confirmed by Sanger sequencing on fish from the F2 generation. Among different lines deleting the Spastin gene, fish exhibiting an insertion of 7 nucleotides at the beginning of exon 2 were used for the present study.

### Locomotion assays on zebrafish larvae

Zebrafish spontaneous activity was monitored at 6 dpf using a Zebrabox recording system (Viewpoint, Lyon, France). One fish was placed per well on a 96 well plate. Recording was performed during 30 min in the dark. Velocity thresholds were set to distinguish between slow and fast velocity (respectively v in green, and V in red) as follow: 3 < v < 6mm/s and V > 6 mm/s [95]. Net velocity was defined as the global larval speed, excluding pausing time. 2μg/ml of Tunicamycin (T7765, Sigma-Aldrich, St. Louis, MO, United States) and 0.2mM of OA (O1383-1G, Sigma Aldrich) [52] were added to the fish water from 5 to 6 dpf and washed prior to the locomotion test.

### RNA extraction and qPCR

Total RNA extraction, RT-PCR and RT-qPCR were performed as previously described [54, 96]. All protocols are detailed in the supporting information in S1 Text. All primers are listed in S2 Table.

### *In situ* hybridization

Probe templates corresponding to the 500 first nucleotides of Spastin M1 (Q6NW58) were obtained by PCR (see primers in S2 Table) and amplicons were flanked with T7 promoter. Sense and anti-sense probes were synthetized by reverse transcription (mMESSAGE mMACHINE T7 Transcription Kit, AM1344, Invitrogen) and purified on Microspin G50 columns (27533001, GE Healthcare, Chicago, IL, United States). *In situ* hybridizations were performed as previously described [95].

### Cloning

CDS were cloned from cDNA obtained from human muscle, brain, or 24 hpf zebrafish embryos and amplified by PCR with ATTB flanking sequences (see primers in S2 Table) to use in the Gateway cloning system. PCR products were purified on agarose gels (MinElute Gel Extraction, 28604, Qiagen, Hilden, Germany) and inserted in Gateways pDon221 vector by recombination using BP clonase II enzyme mix (11789, Invitrogen). For expression in eukaryote cells, CDS were transferred in destination vectors pcDNA-CMV-CherryN or pCI-CMV-3XflagN with LR clonase II enzyme mix (11791, Invitrogen).

### Embryonic cells culture

24 hpf embryos were anesthetized in Tricaine (E10521MS-222, Sigma-Aldrich) and digested in trypsin-EDTA 0.05% (25300, GIBCO, ThermoFischer Scientific). Cell culture was processed at 28°C in L15 complete culture medium (11415–049, GIBCO) as described in Vallone *et al*. [97]. When indicated, 300μM of OA (O1383-1G, Sigma-Aldrich) was added in L15 Medium/2% BSA for 18h prior to fixation.

### Cells transfection and Immunolabeling

HeLa cells were grown in DMEM1X GlutaMAX medium (31966–021, GIBCO) supplemented with 10% FBS (10270–016, GIBCO) and penicillin/streptomycin. Transfection was performed with Lipofectamine 3000 (L3000-01, Invitrogen) following supplier instructions. Immunostaining procedure was performed 48h later. When indicated, 300μM of OA were added in DMEM/2% BSA for 18h before the immunostaining protocol. Similarly, 1μM of nocodazole (M1404, Sigma-Aldrich) and 20μM of Taxol (Paclitaxel, P3456, Invitrogen) were added for 12h prior before the immunolabeling protocol. Cells were fixed 10min in PBS1X/PFA4% then permeabilized in PBX1X/Triton 0.25% for 5min. Primary antibodies were applied in 1% BSA blocking solution for 90min at room temperature. All antibodies and concentrations are listed in the supporting information in S1 Text.

### Confocal imaging and quantification

Image acquisition was performed on a confocal laser-scanning microscope (LSM510, Zeiss, Stockholm, Sweden) at the Cell Imaging Facility of the School of Biology and Medicine. Quantification of LDs number and size was performed with ImageJ (NIH, Bethesda, MA, United States), using the *Analyze Particles* function. For the dispersion analysis, Cartesian coordinates from individual LD (X_LD_;Y_LD_) and from nucleus center (X_Nu_;Y_Nu_) were determined with ImageJ. Distances (d) were calculated using the formula d = √((X_Nu_-X_LD_)^2^ + (Y_Nu_-Y_LD_)^2^).

### Electron microscopy

Electron microscopy was performed at the Electron Microscopy Facility of the School of Biology and Medicine. Zebrafish skeletal muscle were dissected immediately after euthanasia and pre-fixed in PB 0.1M/2.5% glutaraldehyde/PFA4% solution at room temperature for 30min. 1mm cross-sectional sections were cut with a razor blade and fixation was prolonged until 2h. The samples were washed in PB three times (5min distilled water) and dehydrated in acetone solution (Sigma). Careful positioning was performed with binoculars on Aclar film (EMS, Hatfield, PA, United States) inside a Gene Frame (ThermoFischer Scientific) in 65μl of Epon resin and finally polymerized for 48h at 60°C in oven. Sagittal ultrathin sections of 50nm were cut on a Leica Ultracut (Leica Mikrosysteme GmbH, Vienna, Austria) and picked up on a copper slot grid 2x1mm (EMS) coated with a polystyrene film (Sigma). Sections were post stained with 4% uranyl acetate (Sigma). Micrographs were taken with a transmission electron microscope Philips CM100 (ThermoFisher Scientific) with a TVIPS TemCam-F416 digital camera (TVIPS GmbH, Gauting, Germany).

### Seahorse analyzer respirometry

Mitochondrial function was determined with an XFe24 extracellular flux analyzer (Seahorse Bioscience, Billerica, MA, United States). Oxygen consumption rate (OCR) and extracellular acidification rate (ECAR) were measured in dechorionated embryos at 48 hpf. Embryos were staged and placed one per well on an islet capture microplate filled with E3 egg water. The plate was incubated in an incubator without CO_2_ at 28°C for 30min. After measuring baseline OCR as an indication for basal respiration, OCR was measured after an injection of 2μM of FCCP to determine maximal respiration.

### Multi spectral analysis

U2OS cells were prepared for multispectral imaging as previously described [20, 98]. Details are available in the supporting information in S1 Text.

### Lipidomics

Seven months old male zebrafish were anesthetized on ice and killed by decapitation. Brain and skeletal muscle were immediately dissected and flash frozen in liquid nitrogen. Extended protocols for lipid extraction and analyses are described in the supporting information in S1 Text.

## Supporting information

S1 Fig Identification of an uncharacterized splice variant in zebrafish Spastin. (A) Schematic representation of alternative splice suppressing exon 2 to 10 in zebrafish Spastin. (B-C) Confocal microscopy images of zebrafish embryonic cells (B) and HeLa cells (C) overexpressing Spastin splice variant (treated with 300μM oleic acid for 18h before acquisition). Cherry-tagged Spastin appears in red. Tubulin labeling corresponds to microtubules (cyan), Bodipy to LDs (yellow) and Hoechst to nucleus (blue).
(TIF)

S2 Fig Mutations in Spastin M1 and M1Δex4 converge on similar gain of function. (A-B) Confocal microscopy images of HeLa cells overexpressing Spastin splice variants treated with OA for 18h. Cherry-tagged Spastin appears in red. Acetylated Tubulin labeling corresponds to microtubules (cyan), Bodipy to LDs (yellow) and Hoechst to nucleus (blue). In (B) cells were also submitted to 20μM Taxol, 1μM nocodazole (Noco) treatment for 12h, or to cold exposure for 15 min before staining protocol.
(TIF)

S3 Fig Seipin and REEP1 participate to ER/LD dynamics. (A) Schematic representation of human Seipin variant α and β. (B) Confocal microscopy pictures of HeLa cells overexpressing human Seipin α and β with Spastin M1 isoforms (after 18h administration of OA). Cherry-tagged Spastin appears in red, Seipin α and β in green, LDs (Bodipy) in magenta and nucleus (Hoechst) in blue. (C) Confocal microscopy images of HeLa cells overexpressing human REEP1 after 18h administration of OA. REEP1 appears in green, LDs (Bodipy) in magenta and nucleus (Hoechst) in blue. (D) Confocal microscopy images of zebrafish embryonic cells from wild-type and Spastin KO animals (Ctrl and Spa -/-). Cells were transfected with human REEP1 and treated with 300μM oleic acid for 18h. REEP1 labeling (green) was counterstained by bodipy (Magenta).
(TIF)

S4 Fig Spastin influences Atlastin1 pattern and colocalizes with clustered LDs associated with Spartin. (A) Confocal microscopy images of HeLa cells overexpressing human Atlastin1 with Spastin M1 isoforms (after 18h administration of OA). Cherry-tagged Spastin appears in red, Atlastin1 in green, LDs (Bodipy) in magenta and nucleus (Hoechst) in blue. (B) Confocal microscopy images of HeLa cells overexpressing human Spartin with Spastin M1 isoforms (after 18h administration of OA). Cherry-tagged Spastin appears in red, Spartin in green, LDs (Bodipy) in magenta and nucleus (Hoechst) in blue.
(TIF)

S1 Table Quantitative analysis of neutral lipids and phospholipids in brain and muscle from wild-type and Spa-/- zebrafish measured by mass-spectrometry. (A) Unesterified cholesterol (referred as Cholesterol), esterified cholesterol (Chol-C16, Chol-C18 and total) and triacylglycerides (TG) with specific fatty acids composition and carbon total number. (B) Individual and total saturated (SAFA), mono-unsaturated (MUFA) and Poly-unsaturated (PUFA) fatty acids.(C) Individual and total ceramides (Cer), phosphatidylcholines (PC), phosphatidylethanolamines (PE), sphingomyelines (SM) and phosphatidylinositols (PI) with specific fatty acids compositions. All values correspond to lipid quantity per total protein amount. Numbers are mean ± SEM (n = 3 per group). ^#^P<0.08, *P < 0.05, *P* < 0.01, ***P<0.001 (unpaired *t*‐test).
(PDF)

S2 Table List of primers. (PDF)

S1 Text Material and methods. Details of specific sections.
(PDF)

## References

[1] Zhang C, Liu P. The New Face of the Lipid Droplet: Lipid Droplet Proteins. Proteomics. 2018:e1700223 10.1002/pmic.201700223 30216670

[2] Welte MA, Gould AP. Lipid droplet functions beyond energy storage. Biochim Biophys Acta Mol Cell Biol Lipids. 2017;1862(10 Pt B):1260–72. 28735096 10.1016/j.bbalip.2017.07.006 PMC5595650

[3] Vallochi AL, Teixeira L, Oliveira KDS, Maya-Monteiro CM, Bozza PT. Lipid Droplet, a Key Player in Host-Parasite Interactions. Front Immunol. 2018;9:1022 10.3389/fimmu.2018.01022 29875768 PMC5974170

[4] Liu L, MacKenzie KR, Putluri N, Maletic-Savatic M, Bellen HJ. The Glia-Neuron Lactate Shuttle and Elevated ROS Promote Lipid Synthesis in Neurons and Lipid Droplet Accumulation in Glia via APOE/D. Cell Metab. 2017;26(5):719–37 e6. 10.1016/j.cmet.2017.08.024 28965825 PMC5677551

[5] Petan T, Jarc E, Jusovic M. Lipid Droplets in Cancer: Guardians of Fat in a Stressful World. Molecules. 2018;23(8). 10.3390/molecules23081941 PMC6222695 30081476

[6] Dutta A, Sinha DK. Zebrafish lipid droplets regulate embryonic ATP homeostasis to power early development. Open Biol. 2017;7(7). 10.1098/rsob.170063 PMC5541346 28679548

[7] Li X, Li Z, Zhao M, Nie Y, Liu P, Zhu Y, et al Skeletal Muscle Lipid Droplets and the Athlete's Paradox. Cells. 2019;8(3). 10.3390/cells8030249 PMC6468652 30875966

[8] Olzmann JA, Carvalho P. Dynamics and functions of lipid droplets. Nat Rev Mol Cell Biol. 2019;20(3):137–55. 10.1038/s41580-018-0085-z 30523332 PMC6746329

[9] Thiam AR, Beller M. The why, when and how of lipid droplet diversity. J Cell Sci. 2017;130(2):315–24. 10.1242/jcs.192021 28049719

[10] Nettebrock NT, Bohnert M. Born this way—Biogenesis of lipid droplets from specialized ER subdomains. Biochim Biophys Acta Mol Cell Biol Lipids. 2019;In press. 10.1016/j.bbalip.2019.04.008 31028912

[11] Agarwal AK, Garg A. Seipin: a mysterious protein. Trends Mol Med. 2004;10(9):440–4. 10.1016/j.molmed.2004.07.009 15350896

[12] Cartwright BR, Binns DD, Hilton CL, Han S, Gao Q, Goodman JM. Seipin performs dissectible functions in promoting lipid droplet biogenesis and regulating droplet morphology. Mol Biol Cell. 2015;26(4):726–39. 10.1091/mbc.E14-08-1303 25540432 PMC4325842

[13] Gao Q, Binns DD, Kinch LN, Grishin NV, Ortiz N, Chen X, et al Pet10p is a yeast perilipin that stabilizes lipid droplets and promotes their assembly. J Cell Biol. 2017;216(10):3199–217. 10.1083/jcb.201610013 28801319 PMC5626530

[14] Romanauska A, Kohler A. The Inner Nuclear Membrane Is a Metabolically Active Territory that Generates Nuclear Lipid Droplets. Cell. 2018;174(3):700–15 e18. 10.1016/j.cell.2018.05.047 29937227 PMC6371920

[15] Long AP, Manneschmidt AK, VerBrugge B, Dortch MR, Minkin SC, Prater KE, et al Lipid droplet de novo formation and fission are linked to the cell cycle in fission yeast. Traffic. 2012;13(5):705–14. 10.1111/j.1600-0854.2012.01339.x 22300234

[16] Gao G, Chen FJ, Zhou L, Su L, Xu D, Xu L, et al Control of lipid droplet fusion and growth by CIDE family proteins. Biochim Biophys Acta Mol Cell Biol Lipids. 2017;1862(10 Pt B):1197–204. 28648584 10.1016/j.bbalip.2017.06.009

[17] Singh R, Cuervo AM. Lipophagy: connecting autophagy and lipid metabolism. Int J Cell Biol. 2012;2012:282041 10.1155/2012/282041 22536247 PMC3320019

[18] Gao Q, Goodman JM. The lipid droplet-a well-connected organelle. Front Cell Dev Biol. 2015;3:49 10.3389/fcell.2015.00049 26322308 PMC4533013

[19] Salo VT, Ikonen E. Moving out but keeping in touch: contacts between endoplasmic reticulum and lipid droplets. Curr Opin Cell Biol. 2019;57:64–70. 10.1016/j.ceb.2018.11.002 30476754

[20] Valm AM, Cohen S, Legant WR, Melunis J, Hershberg U, Wait E, et al Applying systems-level spectral imaging and analysis to reveal the organelle interactome. Nature. 2017;546(7656):162–7. 10.1038/nature22369 28538724 PMC5536967

[21] Salo VT, Belevich I, Li S, Karhinen L, Vihinen H, Vigouroux C, et al Seipin regulates ER-lipid droplet contacts and cargo delivery. EMBO J. 2016;35(24):2699–716. 10.15252/embj.201695170 27879284 PMC5167346

[22] Wang H, Sreenivasan U, Hu H, Saladino A, Polster BM, Lund LM, et al Perilipin 5, a lipid droplet-associated protein, provides physical and metabolic linkage to mitochondria. J Lipid Res. 2011;52(12):2159–68. 10.1194/jlr.M017939 21885430 PMC3220284

[23] Pu J, Ha CW, Zhang S, Jung JP, Huh WK, Liu P. Interactomic study on interaction between lipid droplets and mitochondria. Protein Cell. 2011;2(6):487–96. 10.1007/s13238-011-1061-y 21748599 PMC4875178

[24] Chang CL, Weigel AV, Ioannou MS, Pasolli HA, Xu CS, Peale DR, et al Spastin tethers lipid droplets to peroxisomes and directs fatty acid trafficking through ESCRT-III. J Cell Biol. 2019;In press. 10.1083/jcb.201902061 PMC6683741 31227594

[25] Welte MA, Gross SP, Postner M, Block SM, Wieschaus EF. Developmental regulation of vesicle transport in Drosophila embryos: forces and kinetics. Cell. 1998;92(4):547–57. 10.1016/s0092-8674(00)80947-2 9491895

[26] Arora GK, Tran SL, Rizzo N, Jain A, Welte MA. Temporal control of bidirectional lipid-droplet motion in Drosophila depends on the ratio of kinesin-1 and its co-factor Halo. J Cell Sci. 2016;129(7):1416–28. 10.1242/jcs.183426 26906417 PMC4852724

[27] Herms A, Bosch M, Reddy BJ, Schieber NL, Fajardo A, Ruperez C, et al AMPK activation promotes lipid droplet dispersion on detyrosinated microtubules to increase mitochondrial fatty acid oxidation. Nat Commun. 2015;6:7176 10.1038/ncomms8176 26013497 PMC4446796

[28] Rai P, Kumar M, Sharma G, Barak P, Das S, Kamat SS, et al Kinesin-dependent mechanism for controlling triglyceride secretion from the liver. Proc Natl Acad Sci U S A. 2017;114(49):12958–63. 10.1073/pnas.1713292114 29158401 PMC5724275

[29] da Silva AF, Mariotti FR, Maximo V, Campello S. Mitochondria dynamism: of shape, transport and cell migration. Cell Mol Life Sci. 2014;71(12):2313–24. 10.1007/s00018-014-1557-8 24442478 PMC11113703

[30] Papadopoulos C, Orso G, Mancuso G, Herholz M, Gumeni S, Tadepalle N, et al Spastin binds to lipid droplets and affects lipid metabolism. PLoS Genet. 2015;11(4):e1005149 10.1371/journal.pgen.1005149 25875445 PMC4395272

[31] Roll-Mecak A, Vale RD. The Drosophila homologue of the hereditary spastic paraplegia protein, spastin, severs and disassembles microtubules. Curr Biol. 2005;15(7):650–5. 10.1016/j.cub.2005.02.029 15823537

[32] Errico A, Ballabio A, Rugarli EI. Spastin, the protein mutated in autosomal dominant hereditary spastic paraplegia, is involved in microtubule dynamics. Hum Mol Genet. 2002;11(2):153–63. 10.1093/hmg/11.2.153 11809724

[33] Evans KJ, Gomes ER, Reisenweber SM, Gundersen GG, Lauring BP. Linking axonal degeneration to microtubule remodeling by Spastin-mediated microtubule severing. J Cell Biol. 2005;168(4):599–606. 10.1083/jcb.200409058 15716377 PMC2171748

[34] Shoukier M, Neesen J, Sauter SM, Argyriou L, Doerwald N, Pantakani DV, et al Expansion of mutation spectrum, determination of mutation cluster regions and predictive structural classification of SPAST mutations in hereditary spastic paraplegia. Eur J Hum Genet. 2009;17(2):187–94. 10.1038/ejhg.2008.147 18701882 PMC2986068

[35] Fink JK. Hereditary spastic paraplegia: clinico-pathologic features and emerging molecular mechanisms. Acta Neuropathol. 2013;126(3):307–28. 10.1007/s00401-013-1115-8 23897027 PMC4045499

[36] Riano E, Martignoni M, Mancuso G, Cartelli D, Crippa F, Toldo I, et al Pleiotropic effects of spastin on neurite growth depending on expression levels. J Neurochem. 2009;108(5):1277–88. 10.1111/j.1471-4159.2009.05875.x 19141076

[37] Claudiani P, Riano E, Errico A, Andolfi G, Rugarli EI. Spastin subcellular localization is regulated through usage of different translation start sites and active export from the nucleus. Exp Cell Res. 2005;309(2):358–69. 10.1016/j.yexcr.2005.06.009 16026783

[38] Fassier C, Tarrade A, Peris L, Courageot S, Mailly P, Dalard C, et al Microtubule-targeting drugs rescue axonal swellings in cortical neurons from spastin knockout mice. Dis Model Mech. 2013;6(1):72–83. 10.1242/dmm.008946 22773755 PMC3529340

[39] Kasher PR, De Vos KJ, Wharton SB, Manser C, Bennett EJ, Bingley M, et al Direct evidence for axonal transport defects in a novel mouse model of mutant spastin-induced hereditary spastic paraplegia (HSP) and human HSP patients. J Neurochem. 2009;110(1):34–44. 10.1111/j.1471-4159.2009.06104.x 19453301

[40] Leo L, Weissmann C, Burns M, Kang M, Song Y, Qiang L, et al Mutant spastin proteins promote deficits in axonal transport through an isoform-specific mechanism involving casein kinase 2 activation. Hum Mol Genet. 2017;26(12):2321–34. 10.1093/hmg/ddx125 28398512 PMC6075366

[41] Fekih R, Tamiru M, Kanzaki H, Abe A, Yoshida K, Kanzaki E, et al The rice (Oryza sativa L.) LESION MIMIC RESEMBLING, which encodes an AAA-type ATPase, is implicated in defense response. Mol Genet Genomics. 2015;290(2):611–22. 10.1007/s00438-014-0944-z 25367283

[42] Song G, Kwon CT, Kim SH, Shim Y, Lim C, Koh HJ, et al The Rice SPOTTED LEAF4 (SPL4) Encodes a Plant Spastin That Inhibits ROS Accumulation in Leaf Development and Functions in Leaf Senescence. Front Plant Sci. 2018;9:1925 10.3389/fpls.2018.01925 30666263 PMC6330318

[43] Matsushita-Ishiodori Y, Yamanaka K, Ogura T. The C. elegans homologue of the spastic paraplegia protein, spastin, disassembles microtubules. Biochem Biophys Res Commun. 2007;359(1):157–62. 10.1016/j.bbrc.2007.05.086 17531954

[44] Chrestian N, Dupre N, Gan-Or Z, Szuto A, Chen S, Venkitachalam A, et al Clinical and genetic study of hereditary spastic paraplegia in Canada. Neurol Genet. 2017;3(1):e122 10.1212/NXG.0000000000000122 27957547 PMC5141523

[45] Lo Giudice T, Lombardi F, Santorelli FM, Kawarai T, Orlacchio A. Hereditary spastic paraplegia: clinical-genetic characteristics and evolving molecular mechanisms. Exp Neurol. 2014;261:518–39. 10.1016/j.expneurol.2014.06.011 24954637

[46] Wood JD, Landers JA, Bingley M, McDermott CJ, Thomas-McArthur V, Gleadall LJ, et al The microtubule-severing protein Spastin is essential for axon outgrowth in the zebrafish embryo. Hum Mol Genet. 2006;15(18):2763–71. 10.1093/hmg/ddl212 16893913

[47] Butler R, Wood JD, Landers JA, Cunliffe VT. Genetic and chemical modulation of spastin-dependent axon outgrowth in zebrafish embryos indicates a role for impaired microtubule dynamics in hereditary spastic paraplegia. Dis Model Mech. 2010;3(11–12):743–51. 10.1242/dmm.004002 20829563 PMC2965401

[48] Jardin N, Giudicelli F, Ten Martin D, Vitrac A, De Gois S, Allison R, et al BMP- and neuropilin 1-mediated motor axon navigation relies on spastin alternative translation. Development. 2018;145(17). 10.1242/dev.162701 PMC6141775 30082270

[49] Svenson IK, Ashley-Koch AE, Pericak-Vance MA, Marchuk DA. A second leaky splice-site mutation in the spastin gene. Am J Hum Genet. 2001;69(6):1407–9. 10.1086/324593 11704932 PMC1235553

[50] Wu M, Neilson A, Swift AL, Moran R, Tamagnine J, Parslow D, et al Multiparameter metabolic analysis reveals a close link between attenuated mitochondrial bioenergetic function and enhanced glycolysis dependency in human tumor cells. Am J Physiol Cell Physiol. 2007;292(1):C125–36. 10.1152/ajpcell.00247.2006 16971499

[51] Koshimizu E, Imamura S, Qi J, Toure J, Valdez DM Jr., Carr CE, et al Embryonic senescence and laminopathies in a progeroid zebrafish model. PLoS One. 2011;6(3):e17688. 21479207 10.1371/journal.pone.0017688 PMC3068137

[52] Holtta-Vuori M, Salo VT, Ohsaki Y, Suster ML, Ikonen E. Alleviation of seipinopathy-related ER stress by triglyceride storage. Hum Mol Genet. 2013;22(6):1157–66. 10.1093/hmg/dds523 23250914

[53] Velazquez AP, Tatsuta T, Ghillebert R, Drescher I, Graef M. Lipid droplet-mediated ER homeostasis regulates autophagy and cell survival during starvation. J Cell Biol. 2016;212(6):621–31. 10.1083/jcb.201508102 26953354 PMC4792078

[54] Li J, Chen Z, Gao LY, Colorni A, Ucko M, Fang S, et al A transgenic zebrafish model for monitoring xbp1 splicing and endoplasmic reticulum stress in vivo. Mech Dev. 2015;137:33–44. 10.1016/j.mod.2015.04.001 25892297

[55] Molon A, Di Giovanni S, Chen YW, Clarkson PM, Angelini C, Pegoraro E, et al Large-scale disruption of microtubule pathways in morphologically normal human spastin muscle. Neurology. 2004;62(7):1097–104. 10.1212/01.wnl.0000118204.90814.5a 15079007

[56] Ochoa CD, Stevens T, Balczon R. Cold exposure reveals two populations of microtubules in pulmonary endothelia. Am J Physiol Lung Cell Mol Physiol. 2011;300(1):L132–8. 10.1152/ajplung.00185.2010 20971804 PMC3023290

[57] Vietri M, Schink KO, Campsteijn C, Wegner CS, Schultz SW, Christ L, et al Spastin and ESCRT-III coordinate mitotic spindle disassembly and nuclear envelope sealing. Nature. 2015;522(7555):231–5. 10.1038/nature14408 26040712

[58] Lumb JH, Connell JW, Allison R, Reid E. The AAA ATPase spastin links microtubule severing to membrane modelling. Biochim Biophys Acta. 2012;1823(1):192–7. 10.1016/j.bbamcr.2011.08.010 21888932

[59] Plaud C, Joshi V, Kajevu N, Pous C, Curmi PA, Burgo A. Functional differences of short and long isoforms of spastin harboring missense mutation. Dis Model Mech. 2018;11(9). 10.1242/dmm.033704 PMC6177001 30213879

[60] Wang S, Tukachinsky H, Romano FB, Rapoport TA. Cooperation of the ER-shaping proteins atlastin, lunapark, and reticulons to generate a tubular membrane network. Elife. 2016;5. 10.7554/eLife.18605 PMC5021524 27619977

[61] Moss TJ, Andreazza C, Verma A, Daga A, McNew JA. Membrane fusion by the GTPase atlastin requires a conserved C-terminal cytoplasmic tail and dimerization through the middle domain. Proc Natl Acad Sci U S A. 2011;108(27):11133–8. 10.1073/pnas.1105056108 21690399 PMC3131361

[62] Yalcin B, Zhao L, Stofanko M, O'Sullivan NC, Kang ZH, Roost A, et al Modeling of axonal endoplasmic reticulum network by spastic paraplegia proteins. Elife. 2017;6. 10.7554/eLife.23882 PMC5576921 28742022

[63] Beetz C, Koch N, Khundadze M, Zimmer G, Nietzsche S, Hertel N, et al A spastic paraplegia mouse model reveals REEP1-dependent ER shaping. J Clin Invest. 2013;123(10):4273–82. 10.1172/JCI65665 24051375 PMC3784524

[64] Park SH, Zhu PP, Parker RL, Blackstone C. Hereditary spastic paraplegia proteins REEP1, spastin, and atlastin-1 coordinate microtubule interactions with the tubular ER network. J Clin Invest. 2010;120(4):1097–110. 10.1172/JCI40979 20200447 PMC2846052

[65] Eastman SW, Yassaee M, Bieniasz PD. A role for ubiquitin ligases and Spartin/SPG20 in lipid droplet turnover. J Cell Biol. 2009;184(6):881–94. 10.1083/jcb.200808041 19307600 PMC2699154

[66] Falk J, Rohde M, Bekhite MM, Neugebauer S, Hemmerich P, Kiehntopf M, et al Functional mutation analysis provides evidence for a role of REEP1 in lipid droplet biology. Hum Mutat. 2014;35(4):497–504. 10.1002/humu.22521 24478229

[67] Klemm RW, Norton JP, Cole RA, Li CS, Park SH, Crane MM, et al A conserved role for atlastin GTPases in regulating lipid droplet size. Cell Rep. 2013;3(5):1465–75. 10.1016/j.celrep.2013.04.015 23684613 PMC3742324

[68] Zelnik ID, Ventura AE, Kim JL, Silva LC, Futerman AH. The role of ceramide in regulating endoplasmic reticulum function. Biochim Biophys Acta Mol Cell Biol Lipids. 2019. 10.1016/j.bbalip.2019.06.015 31233888

[69] Patel D, Witt SN. Ethanolamine and Phosphatidylethanolamine: Partners in Health and Disease. Oxid Med Cell Longev. 2017;2017:4829180 10.1155/2017/4829180 28785375 PMC5529665

[70] Lareau LF, Green RE, Bhatnagar RS, Brenner SE. The evolving roles of alternative splicing. Curr Opin Struct Biol. 2004;14(3):273–82. 10.1016/j.sbi.2004.05.002 15193306

[71] Arendt T, Stieler JT, Holzer M. Tau and tauopathies. Brain Res Bull. 2016;126(Pt 3):238–92. 10.1016/j.brainresbull.2016.08.018 27615390

[72] Matsushita-Ishiodori Y, Yamanaka K, Hashimoto H, Esaki M, Ogura T. Conserved aromatic and basic amino acid residues in the pore region of Caenorhabditis elegans spastin play critical roles in microtubule severing. Genes Cells. 2009;14(8):925–40. 10.1111/j.1365-2443.2009.01320.x 19619244

[73] Salinas S, Carazo-Salas RE, Proukakis C, Cooper JM, Weston AE, Schiavo G, et al Human spastin has multiple microtubule-related functions. J Neurochem. 2005;95(5):1411–20. 10.1111/j.1471-4159.2005.03472.x 16219033

[74] Svenson IK, Ashley-Koch AE, Gaskell PC, Riney TJ, Cumming WJ, Kingston HM, et al Identification and expression analysis of spastin gene mutations in hereditary spastic paraplegia. Am J Hum Genet. 2001;68(5):1077–85. 10.1086/320111 11309678 PMC1226088

[75] Solowska JM, Morfini G, Falnikar A, Himes BT, Brady ST, Huang D, et al Quantitative and functional analyses of spastin in the nervous system: implications for hereditary spastic paraplegia. J Neurosci. 2008;28(9):2147–57. 10.1523/JNEUROSCI.3159-07.2008 18305248 PMC2693295

[76] Hamada T, Ueda H, Kawase T, Hara-Nishimura I. Microtubules contribute to tubule elongation and anchoring of endoplasmic reticulum, resulting in high network complexity in Arabidopsis. Plant Physiol. 2014;166(4):1869–76. 10.1104/pp.114.252320 25367857 PMC4256883

[77] Onal G, Kutlu O, Gozuacik D, Dokmeci Emre S. Lipid Droplets in Health and Disease. Lipids Health Dis. 2017;16(1):128 10.1186/s12944-017-0521-7 28662670 PMC5492776

[78] Solowska JM, Garbern JY, Baas PW. Evaluation of loss of function as an explanation for SPG4-based hereditary spastic paraplegia. Hum Mol Genet. 2010;19(14):2767–79. 10.1093/hmg/ddq177 20430936 PMC2893808

[79] Solowska JM D 'Rozario M, Jean DC, Davidson MW, Marenda DR, Baas PW. Pathogenic mutation of spastin has gain-of-function effects on microtubule dynamics. J Neurosci. 2014;34(5):1856–67. 10.1523/JNEUROSCI.3309-13.2014 24478365 PMC3905148

[80] Johnson MR, Stephenson RA, Ghaemmaghami S, Welte MA. Developmentally regulated H2Av buffering via dynamic sequestration to lipid droplets in Drosophila embryos. Elife. 2018;7. 10.7554/eLife.36021 PMC6089599 30044219

[81] Winsor J, Machi U, Han Q, Hackney DD, Lee TH. GTP hydrolysis promotes disassembly of the atlastin crossover dimer during ER fusion. J Cell Biol. 2018;217(12):4184–98. 10.1083/jcb.201805039 30249723 PMC6279388

[82] Yao L, Xie D, Geng L, Shi D, Huang J, Wu Y, et al REEP5 (Receptor Accessory Protein 5) Acts as a Sarcoplasmic Reticulum Membrane Sculptor to Modulate Cardiac Function. J Am Heart Assoc. 2018;7(3). 10.1161/JAHA.117.007205 PMC5850239 29431104

[83] Lee M, Paik SK, Lee MJ, Kim YJ, Kim S, Nahm M, et al Drosophila Atlastin regulates the stability of muscle microtubules and is required for synapse development. Dev Biol. 2009;330(2):250–62. 10.1016/j.ydbio.2009.03.019 19341724

[84] Pennings M, Schouten MI, van Gaalen J, Meijer RPP, de Bot ST, Kriek M, et al KIF1A variants are a frequent cause of autosomal dominant hereditary spastic paraplegia. Eur J Hum Genet. 2020;28(1):40–9. 10.1038/s41431-019-0497-z 31488895 PMC6906463

[85] Guardia CM, Farias GG, Jia R, Pu J, Bonifacino JS. BORC Functions Upstream of Kinesins 1 and 3 to Coordinate Regional Movement of Lysosomes along Different Microtubule Tracks. Cell Rep. 2016;17(8):1950–61. 10.1016/j.celrep.2016.10.062 27851960 PMC5136296

[86] Wozniak MJ, Bola B, Brownhill K, Yang YC, Levakova V, Allan VJ. Role of kinesin-1 and cytoplasmic dynein in endoplasmic reticulum movement in VERO cells. J Cell Sci. 2009;122(Pt 12):1979–89. 10.1242/jcs.041962 19454478 PMC2723153

[87] Vega AL, Yuan C, Votaw VS, Santana LF. Dynamic changes in sarcoplasmic reticulum structure in ventricular myocytes. J Biomed Biotechnol. 2011;2011:382586 10.1155/2011/382586 22131804 PMC3206393

[88] Lu J, Rashid F, Byrne PC. The hereditary spastic paraplegia protein spartin localises to mitochondria. J Neurochem. 2006;98(6):1908–19. 10.1111/j.1471-4159.2006.04008.x 16945107

[89] Renvoise B, Malone B, Falgairolle M, Munasinghe J, Stadler J, Sibilla C, et al Reep1 null mice reveal a converging role for hereditary spastic paraplegia proteins in lipid droplet regulation. Hum Mol Genet. 2016;25(23):5111–25. 10.1093/hmg/ddw315 27638887 PMC6078631

[90] Montenegro G, Rebelo AP, Connell J, Allison R, Babalini C, D'Aloia M, et al Mutations in the ER-shaping protein reticulon 2 cause the axon-degenerative disorder hereditary spastic paraplegia type 12. J Clin Invest. 2012;122(2):538–44. 10.1172/JCI60560 22232211 PMC3266795

[91] Hashimoto Y, Shirane M, Matsuzaki F, Saita S, Ohnishi T, Nakayama KI. Protrudin regulates endoplasmic reticulum morphology and function associated with the pathogenesis of hereditary spastic paraplegia. J Biol Chem. 2014;289(19):12946–61. 10.1074/jbc.M113.528687 24668814 PMC4036311

[92] Yamamoto Y, Yoshida A, Miyazaki N, Iwasaki K, Sakisaka T. Arl6IP1 has the ability to shape the mammalian ER membrane in a reticulon-like fashion. Biochem J. 2014;458(1):69–79. 10.1042/BJ20131186 24262037

[93] Mancuso G, Barth E, Crivello P, Rugarli EI. Alternative splicing of Spg7, a gene involved in hereditary spastic paraplegia, encodes a variant of paraplegin targeted to the endoplasmic reticulum. PLoS One. 2012;7(5):e36337 10.1371/journal.pone.0036337 22563492 PMC3341365

[94] Kimmel CB, Ballard WW, Kimmel SR, Ullmann B, Schilling TF. Stages of embryonic development of the zebrafish. Dev Dyn. 1995;203(3):253–310. 10.1002/aja.1002030302 8589427

[95] Arribat Y, Mysiak KS, Lescouzeres L, Boizot A, Ruiz M, Rossel M, et al Sonic Hedgehog repression underlies gigaxonin mutation-induced motor deficits in giant axonal neuropathy. J Clin Invest. 2019. 10.1172/JCI129788 PMC6877328 31503551

[96] Arribat Y, Broskey NT, Greggio C, Boutant M, Conde Alonso S, Kulkarni SS, et al Distinct patterns of skeletal muscle mitochondria fusion, fission and mitophagy upon duration of exercise training. Acta Physiol (Oxf). 2019;225(2):e13179. 30144291 10.1111/apha.13179

[97] Vallone D, Santoriello C, Gondi SB, Foulkes NS. Basic protocols for zebrafish cell lines: maintenance and transfection. Methods Mol Biol. 2007;362:429–41. 10.1007/978-1-59745-257-1_35 17417032

[98] Cohen S, Valm AM, Lippincott-Schwartz J. Multispectral Live-Cell Imaging. Current protocols in cell biology. 2018;79(1):e46 10.1002/cpcb.46 29924484 PMC6283277

