# Supplementary figures and images for: Spastin mutations impair coordination between lipid droplet dispersion and reticulum
Source: PLoS Genet. 2020 Apr 21;16(4):e1008665. doi: 10.1371/journal.pgen.1008665 (PMC7173978; doi:10.1371/journal.pgen.1008665)

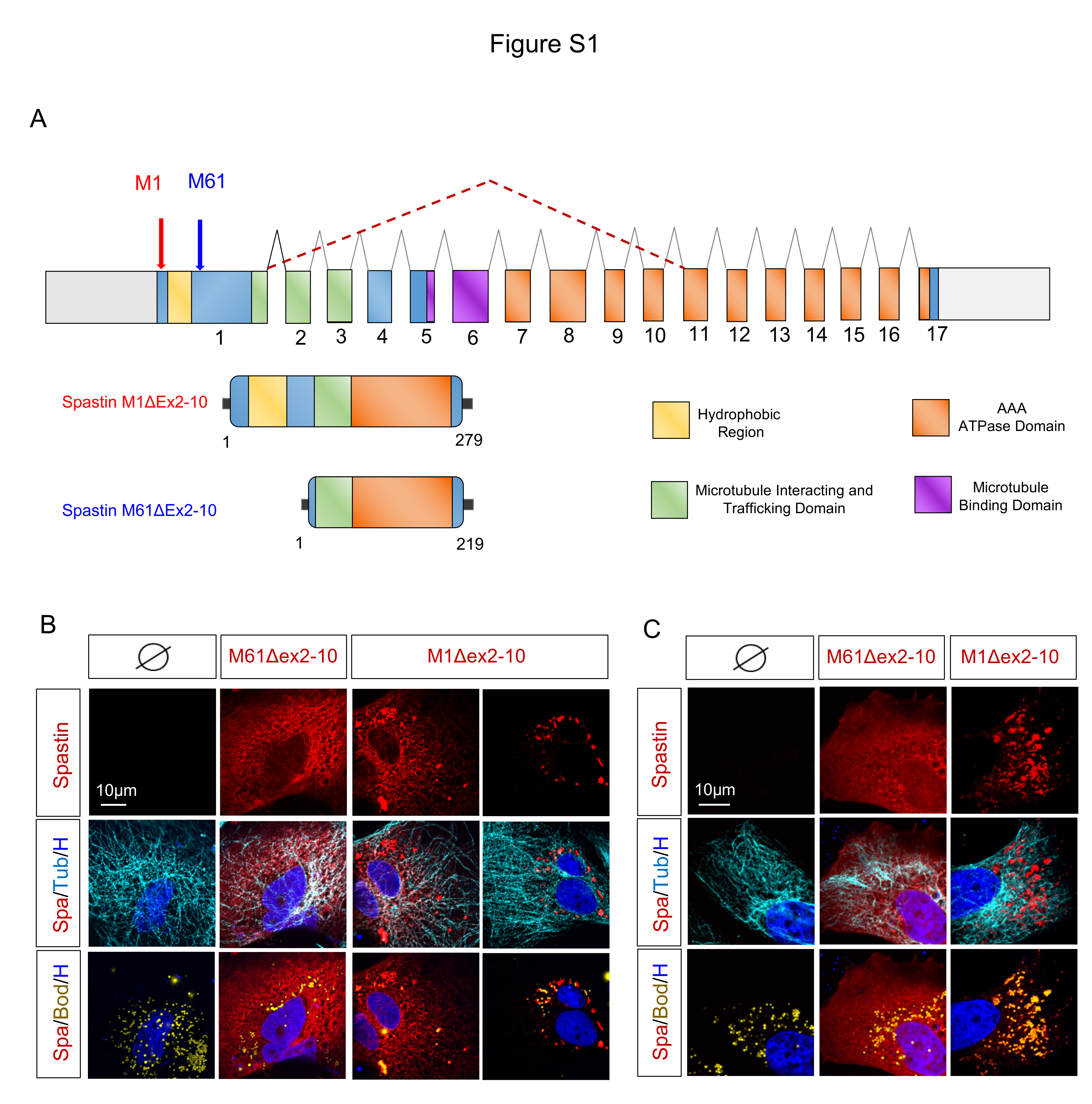

Supplement: S1 Fig — (A) Schematic representation of alternative splice suppressing exon 2 to 10 in zebrafish Spastin. (B-C) Confocal microscopy images of zebrafish embryonic cells (B) and HeLa cells (C) overexpressing Spastin splice variant (treated with 300μM oleic acid for 18h before acquisition). Cherry-tagged Spastin appears in red. Tubulin labeling corresponds to microtubules (cyan), Bodipy to LDs (yellow) and Hoechst to nucleus (blue). (TIF) [file pgen.1008665.s001.tif]

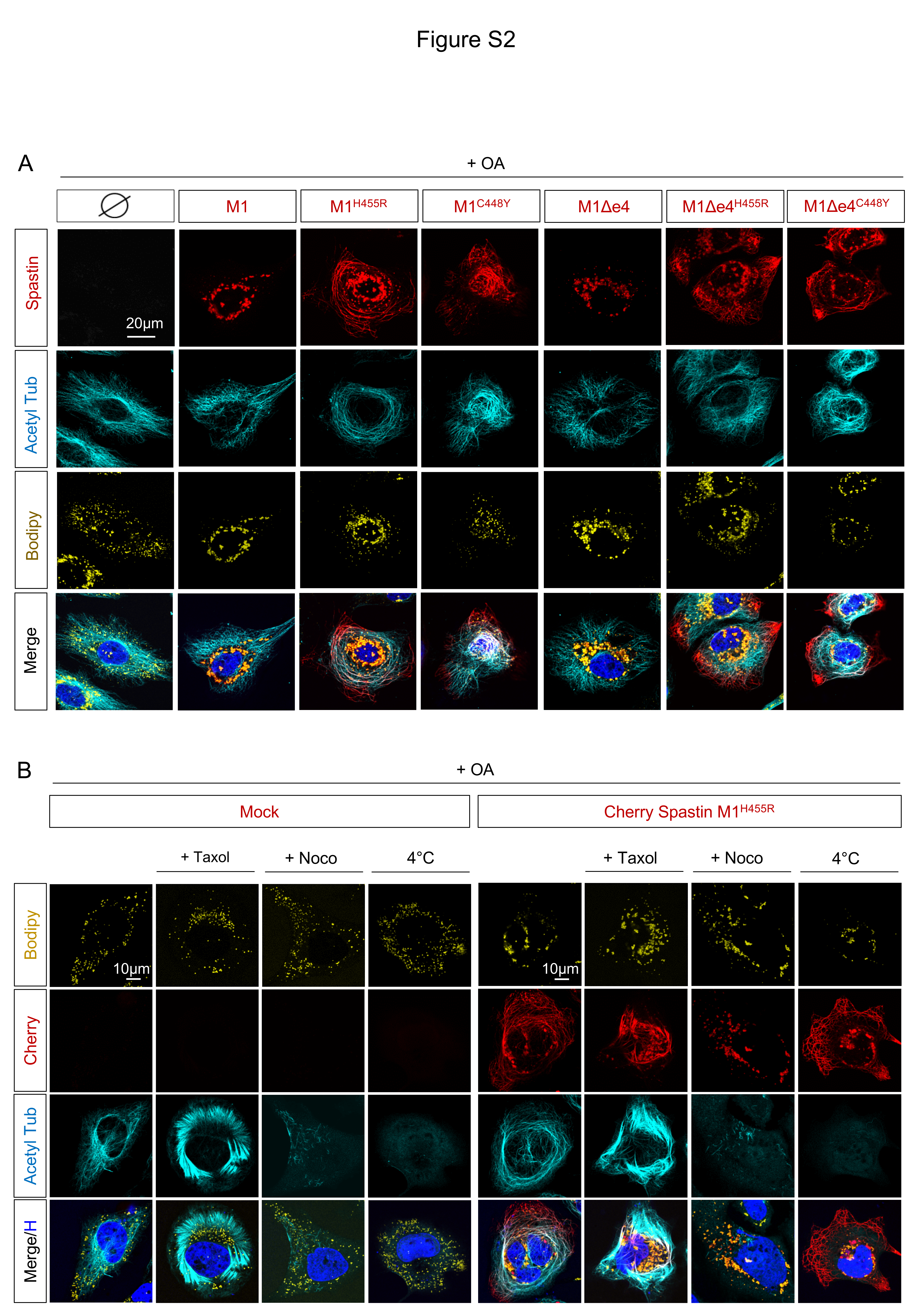

Supplement: S2 Fig — (A-B) Confocal microscopy images of HeLa cells overexpressing Spastin splice variants treated with OA for 18h. Cherry-tagged Spastin appears in red. Acetylated Tubulin labeling corresponds to microtubules (cyan), Bodipy to LDs (yellow) and Hoechst to nucleus (blue). In (B) cells were also submitted to 20μM Taxol, 1μM nocodazole (Noco) treatment for 12h, or to cold exposure for 15 min before staining protocol. (TIF) [file pgen.1008665.s002.tif]

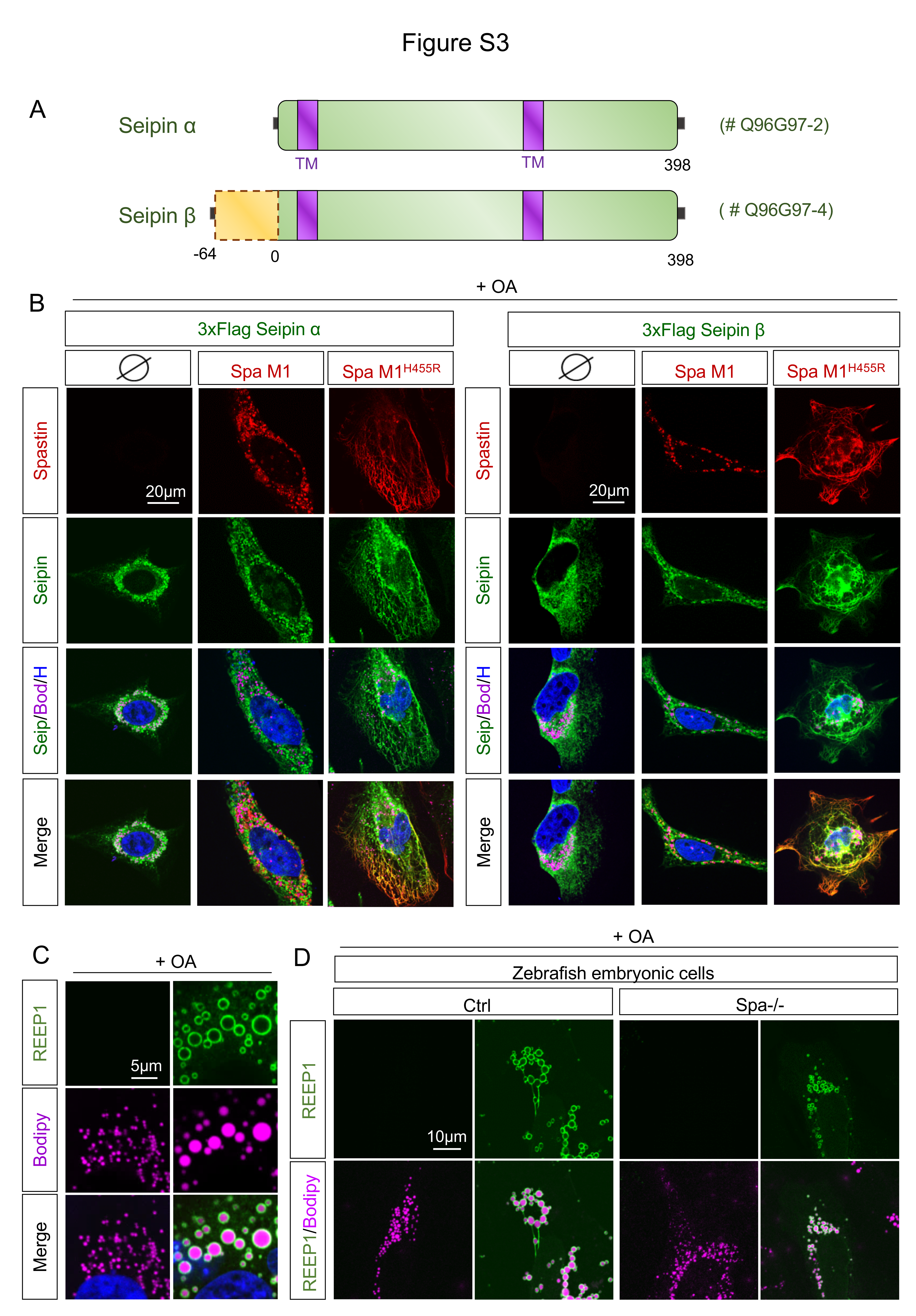

Supplement: S3 Fig — (A) Schematic representation of human Seipin variant α and β. (B) Confocal microscopy pictures of HeLa cells overexpressing human Seipin α and β with Spastin M1 isoforms (after 18h administration of OA). Cherry-tagged Spastin appears in red, Seipin α and β in green, LDs (Bodipy) in magenta and nucleus (Hoechst) in blue. (C) Confocal microscopy images of HeLa cells overexpressing human REEP1 after 18h administration of OA. REEP1 appears in green, LDs (Bodipy) in magenta and nucleus (Hoechst) in blue. (D) Confocal microscopy images of zebrafish embryonic cells from wild-type and Spastin KO animals (Ctrl and Spa -/-). Cells were transfected with human REEP1 and treated with 300μM oleic acid for 18h. REEP1 labeling (green) was counterstained by bodipy (Magenta). (TIF) [file pgen.1008665.s003.tif]

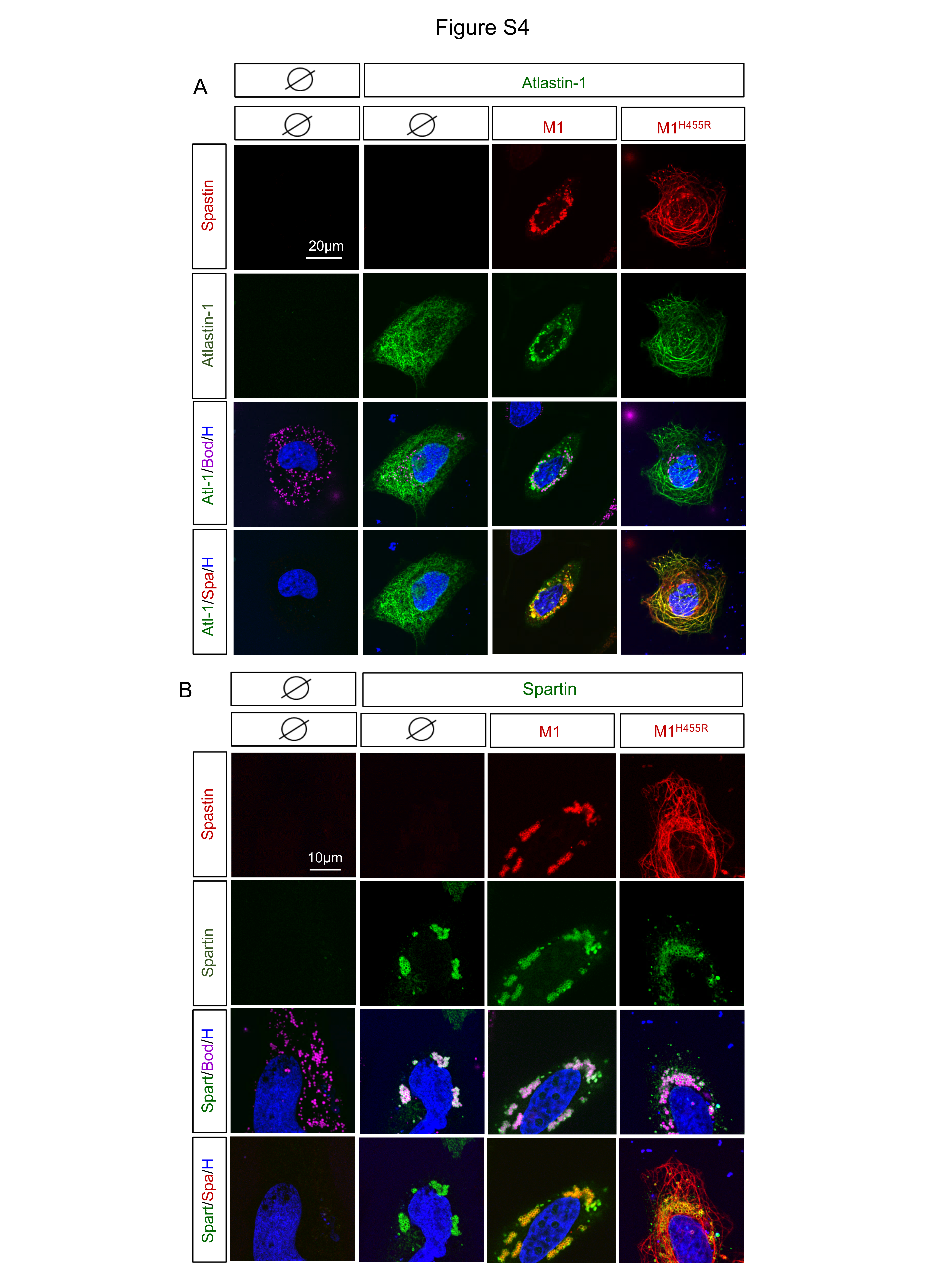

Supplement: S4 Fig — (A) Confocal microscopy images of HeLa cells overexpressing human Atlastin1 with Spastin M1 isoforms (after 18h administration of OA). Cherry-tagged Spastin appears in red, Atlastin1 in green, LDs (Bodipy) in magenta and nucleus (Hoechst) in blue. (B) Confocal microscopy images of HeLa cells overexpressing human Spartin with Spastin M1 isoforms (after 18h administration of OA). Cherry-tagged Spastin appears in red, Spartin in green, LDs (Bodipy) in magenta and nucleus (Hoechst) in blue. (TIF) [file pgen.1008665.s004.tif]
