# Supplementary material for: Spastin mutations impair coordination between lipid droplet dispersion and reticulum
Source: PLoS Genet. 2020 Apr 21;16(4):e1008665. doi: 10.1371/journal.pgen.1008665 (PMC7173978; doi:10.1371/journal.pgen.1008665)
Supplement: S1 Table — (A) Unesterified cholesterol (referred as Cholesterol), esterified cholesterol (Chol-C16, Chol-C18 and total) and triacylglycerides (TG) with specific fatty acids composition and carbon total number. (B) Individual and total saturated (SAFA), mono-unsaturated (MUFA) and Poly-unsaturated (PUFA) fatty acids.(C) Individual and total ceramides (Cer), phosphatidylcholines (PC), phosphatidylethanolamines (PE), sphingomyelines (SM) and phosphatidylinositols (PI) with specific fatty acids compositions. All values correspond to lipid quantity per total protein amount. Numbers are mean ± SEM (n = 3 per group). #P<0.08, *P < 0.05, P < 0.01, ***P<0.001 (unpaired t‐test). (PDF) [file pgen.1008665.s005.pdf]

Table S1

A

		Brain				Muscle				Respective Student *t* test *P* value	
		Ctrl		Spa-/-		Ctrl		Spa-/-			
		Mean	SEM	Mean	SEM	Mean	SEM	Mean	SEM	Brain Ctrl vs Spa-/-	Muscle Ctrl vs Spa-/-
**SAFA**	C16:0	7.218	0.794	6.832	1.031	1.314	0.098	0.857*	0.089	0.782	0.026
	C18:0	7.689	0.472	7.720	0.297	0.627	0.095	0.604	0.017	0.958	0.829
	C20:0					0.024	0.007	0.025	0.002		0.901
	C24:0	0.971	0.027	0.992	0.076					0.810	
**MUFA**	C16:1ω9	0.929	0.127	0.940	0.042	0.029	0.002	0.016#	0.005	0.941	0.068
	C16:1ω7	0.472	0.080	0.430	0.063	0.038	0.003	0.021#	0.006	0.700	0.053
	C18:1ω9c	10.805	0.968	10.913	0.552	0.531	0.055	0.419	0.015	0.927	0.122
	C18:1ω9t	0.406	0.130	0.670	0.024	0.108	0.022	0.106	0.001	0.117	0.943
	C20:1ω9					0.143	0.033	0.161	0.013		0.632
	C24:1ω9	0.572	0.074	0.595	0.062	0.179	0.036	0.170	0.025	0.822	0.841
**PUFA**	C16:2ω4	0.581	0.038	0.939	0.205	0.158	0.002	0.048*	0.017	0.161	0.003
	C16:3	29.687	2.609	36.370	2.235	0.304	0.017	0.149*	0.024	0.124	0.006
	C18:2ω6	1.606	0.150	1.861	0.042	0.522	0.095	0.515	0.016	0.176	0.941
	C18:3ω6	0.407	0.056	0.396	0.019	0.023	0.005	0.019	0.003	0.863	0.553
	C18:3ω3	1.338	0.305	2.010	0.143	0.036	0.004	0.030	0.002	0.116	0.316
	C20:2ω6	0.197	0.063	0.214	0.021	0.026	0.006	0.021	0.001	0.802	0.405
	C20:3ω6	0.140	0.021	0.154	0.026	0.012	0.006	0.007	0.000	0.708	0.446
	C20:4ω6	2.555	0.184	2.606	0.160	0.121	0.021	0.110	0.001	0.843	0.611
	C20:5ω3	1.977	0.441	1.652	0.142	0.215	0.036	0.200	0.001	0.522	0.700
	C22:4ω6	0.618	0.096	0.640	0.021	0.043	0.012	0.028	0.003	0.833	0.261
	C22:5ω3	0.463	0.014	0.406	0.021	0.089	0.018	0.078	0.006	0.087	0.610
	C22:6ω3	10.669	0.502	10.730	0.430	1.475	0.335	1.455	0.019	0.932	0.955
**Saturated**		15.878	1.163	15.544	0.939	1.965	0.198	1.486	0.095	0.834	0.095
**Mono-insaturated**		13.183	1.138	13.547	0.641	1.028	0.147	0.893	0.044	0.794	0.428
**Poly-insaturated**		50.237	3.080	57.977	3.201	3.025	0.540	2.660	0.077	0.156	0.540
**Total**		79.298	4.802	87.069	4.085	6.017	0.877	5.039	0.204	0.285	0.338

B

	Brain				Muscle				Student test	
	Ctrl		Spa-/-		Ctrl		Spa-/-			
	Mean	SEM	Mean	SEM	Mean	SEM	Mean	SEM	Brain Ctrl vs Spa-/-	Muscle Ctrl vs Spa-/-
**Cholesterol**	56.844	4.746	60.343	1.579	2.303	0.227	1.725#	0.080	0.523	0.074
Chol-C16	nd	nd	nd	nd	0.080	0.021	0.043	0.004		0.159
Chol-C18	nd	nd	nd	nd	0.186	0.017	0.104*	0.003		0.008
**Total Est Chol**	nd	nd	nd	nd	0.266	0.013	0.147*	0.005		0.001
C49-TG (14/16/16)	nd	nd	nd	nd	0.546	0.116	0.146*	0.049		0.034
C51-TG (16/16/16)	0.368	0.028	0.446	0.207	3.193	0.574	1.012*	0.297	0.659	0.028
C53-TG (16/16/18)	1.756	0.118	1.607	0.823	9.452	1.869	3.667#	0.993	0.867	0.052
C55-TG (16/18/18)	2.948	0.154	2.935	1.551	12.727	2.722	5.541#	1.405	0.994	0.079
C57-TG (18/18/18)	2.106	0.111	2.294	1.202	7.780	1.787	3.441	0.812	0.884	0.092
C59-TG (18/18/20)	0.374	0.029	0.389	0.246	2.387	0.526	1.072	0.263	0.957	0.089
**Total TG**	7.553	0.435	7.522	4.012	36.085	7.461	14.878#	3.816	0.994	0.065
**Total NL**	64.397	4.316	67.865	2.573	38.654	7.683	16.751#	3.890	0.528	0.064

C

		Brain				Muscle				Student test	
		Ctrl		Spa-/-		Ctrl		Spa-/-		Brain Ctrl vs Spa-/-	Muscle Ctrl vs Spa-/-
		Mean	SEM	Mean	SEM	Mean	SEM	Mean	SEM		
**Cer**	Cer d18:1/16_0	3.311	0.235	2.267	0.484	0.760	0.231	0.561	0.075	0.124	0.458
	Cer d18:1/18:0	10.487	0.978	7.529*	0.248	0.038	0.007	0.024	0.003	0.043	0.133
	Cer d18:1/20:0	0.993	0.081	1.010	0.044	0.038	0.012	0.023	0.001	0.864	0.287
	Cer d18:1/22:0	9.119	1.064	10.240	0.249	0.129	0.011	0.068*	0.008	0.363	0.011
	Cer d18:1/24:0	20.896	2.185	20.810	1.073	0.663	0.096	0.418	0.040	0.973	0.078
	Cer d18:1/24:1	14.634	1.913	15.031	0.490	0.548	0.087	0.399	0.037	0.850	0.190
	Cer d18:1/26:0	1.739	0.119	1.788	0.207	0.093	0.016	0.052#	0.003	0.848	0.075
	Cer d18:1/26:1	2.084	0.121	2.092	0.292	0.113	0.019	0.061#	0.003	0.981	0.052
	**Total Cer**	63.263	6.132	60.767	1.473	2.382	0.373	1.607	0.154	0.712	0.128
**PC**	PC28:0	0.467	0.094	0.539	0.054	0.073	0.012	0.061	0.002	0.544	0.394
	PC30:0	28.927	4.114	33.825	2.977	2.396	0.271	2.534	0.222	0.389	0.715
	PC30:1	2.606	0.216	3.359#	0.228	0.380	0.119	0.415	0.029	0.074	0.792
	PC32:0	797.964	103.598	1072.857	113.992	33.948	7.400	37.786	4.404	0.149	0.679
	PC32:1	138.487	34.894	194.801	35.386	5.851	1.340	5.806	0.545	0.320	0.977
	PC32:2	7.125	1.231	11.886	1.948	0.622	0.146	0.738	0.081	0.108	0.526
	PC34:0	302.382	60.189	443.347	78.403	10.780	2.033	12.720	1.025	0.227	0.442
	PC34:1	2257.603	515.734	3125.793	551.501	78.857	16.692	89.072	7.074	0.314	0.603
	PC34:2	170.364	44.014	280.255	52.777	38.984	8.709	54.449	2.589	0.185	0.164
	PC34:3	14.520	3.575	23.919	3.616	2.234	0.489	3.118	0.183	0.138	0.166
	PC36:1	680.319	135.087	1026.926	196.113	20.288	4.378	28.666	1.790	0.219	0.151
	PC36:2	174.682	41.550	289.778	62.017	12.306	2.711	15.994	1.018	0.198	0.272
	PC36:3	43.693	12.593	69.569	13.079	10.931	2.644	15.630	0.753	0.227	0.163
	PC36:4	42.139	10.646	58.045	6.525	10.396	2.419	15.049	1.082	0.272	0.154
	PC38:2	68.034	13.911	109.263	23.167	2.109	0.542	3.387	0.227	0.202	0.095
	PC38:3	21.483	5.454	32.935	5.504	2.736	0.698	4.177	0.243	0.213	0.123
	PC38:4	39.890	9.457	51.568	5.263	3.712	0.898	4.609	0.143	0.341	0.380
	PC38:5	174.018	42.580	233.986	36.994	18.262	4.014	24.053	2.104	0.348	0.270
	PC38:6	980.405	235.988	1371.361	240.577	73.954	15.794	103.834	13.526	0.311	0.224
	PC40:3	28.435	6.529	48.519	10.650	0.364	0.084	0.673#	0.089	0.183	0.065
	PC40:6	407.301	72.280	529.827	56.646	18.207	3.774	25.875	2.459	0.253	0.164
	**Total PC**	6380.842	1347.258	9012.356	1493.600	347.392	71.925	448.648	38.115	0.261	0.281
	PE32:0	2.478	0.319	3.448#	0.252	0.093	0.012	0.086	0.008	0.075	0.619
	PE32:1	1.694	0.203	2.379	0.230	0.061	0.009	0.052	0.003	0.089	0.381

PE	PE34:0	26.756	2.250	36.017*	2.406	1.163	0.116	1.048	0.063	0.048	0.434
	PE34:1	5.980	0.857	8.543#	0.656	0.401	0.058	0.414	0.015	0.076	0.842
	PE36:1	46.716	1.317	60.546*	2.776	3.082	0.540	3.078	0.145	0.011	0.996
	PE36:2	14.488	1.305	20.884*	1.546	1.404	0.224	1.507	0.046	0.034	0.674
	PE36:3	5.476	0.933	8.059	0.755	0.733	0.116	0.813	0.022	0.098	0.536
	PE36:4	3.021	0.593	4.035	0.424	0.439	0.079	0.487	0.022	0.236	0.591
	PE38:2	5.191	0.682	6.296	0.594	0.532	0.119	0.519	0.007	0.289	0.918
	PE38:3	12.114	1.667	15.046	0.828	1.706	0.294	1.938	0.088	0.190	0.490
	PE38:4	15.473	2.319	19.647	1.823	2.432	0.458	2.613	0.090	0.230	0.718
	PE38:5	56.499	8.732	72.128	7.426	3.854	0.802	4.013	0.432	0.244	0.869
	PE38:6	99.722	18.803	134.666	17.142	6.595	1.390	6.998	0.674	0.242	0.807
	PE40:3	4.404	0.222	5.734*	0.380	0.411	0.090	0.450	0.024	0.039	0.703
	PE40:5	402.640	64.147	525.824	41.413	28.268	6.446	37.517	2.730	0.182	0.257
	PE40:6	213.375	28.221	278.489	23.704	18.616	4.787	23.464	1.686	0.152	0.393
	PE40:7	20.946	2.951	28.802#	1.122	6.515	2.091	7.885	0.086	0.068	0.548
	**Total PE**	936.973	134.729	1230.544	101.329	76.304	17.399	92.883	5.510	0.157	0.415
SM	SM d18:1/14:0	13.848	2.932	18.324	0.369	5.705	1.548	5.758	0.991	0.204	0.978
	SM d18:1/16:0	400.951	23.185	413.808	13.821	61.229	5.998	36.565*	6.127	0.659	0.045
	SM d18:1/16:1	0.000	0.000	0.000	0.000	0.700	0.153	0.801	0.146		0.658
	SM d18:1/18:0	388.146	32.231	455.013	61.430	1.545	0.236	0.920	0.185	0.390	0.105
	SM d18:1/18:1	0.000	0.000	0.000	0.000	0.437	0.120	0.393	0.082		0.780
	SM d18:1/20:0	67.837	8.131	82.354	7.445	4.459	0.554	3.426	0.446	0.258	0.220
	SM d18:1/20:1	2.283	2.283	5.070	2.549	0.859	0.299	0.766	0.151	0.461	0.796
	SM d18:1/22:0	277.781	45.499	309.104	21.076	8.839	1.709	5.738	0.790	0.566	0.175
	SM d18:1/22:1	76.723	14.225	96.609	8.134	13.783	3.693	13.800	2.403	0.292	0.997
	SM d18:1/24:0	436.328	78.428	517.728	38.429	12.273	2.883	6.629	0.703	0.404	0.130
	SM d18:1/24:1	908.975	139.843	1146.070	69.147	41.404	8.637	27.745	3.081	0.203	0.211
	**Total SM**	2572.872	340.273	3044.081	176.047	151.232	20.175	102.541	14.892	0.286	0.124
PI	PI34:1	8.480	0.978	9.152	0.299	0.892	0.133	0.692	0.044	0.547	0.226
	PI34:2	5.085	2.585	7.175	0.800	0.818	0.112	0.717	0.026	0.483	0.430
	PI36:1	13.379	2.246	15.342	1.004	2.895	0.213	3.346	0.434	0.470	0.403
	PI36:2	20.269	4.272	24.730	1.343	6.517	0.690	7.215	0.739	0.376	0.528
	PI36:3	21.572	1.151	24.759	1.870	4.173	0.539	3.924	0.457	0.220	0.743
	PI38:1	1.455	1.455	0.000	0.000	0.000	0.000	0.000	0.000	0.374	
	PI38:2	16.968	0.410	20.483*	0.712	2.387	0.311	1.949	0.207	0.013	0.306
	PI38:3	168.473	15.334	152.943	1.708	32.138	4.457	27.760	2.626	0.371	0.445
	PI38:4	421.773	13.295	398.324	14.058	84.220	10.807	79.319	1.633	0.292	0.677
	PI38:5	277.248	8.893	255.669	15.066	56.148	6.137	49.441	3.433	0.285	0.394
	PI40:3	0.386	0.386	0.000	0.000	0.000	0.000	0.000	0.000	0.374	
	PI40:4	21.565	2.154	21.561	0.542	5.897	0.998	5.540	0.079	0.999	0.739
	PI40:5	104.222	9.297	105.964	6.880	28.680	5.823	29.190	1.204	0.888	0.936
	PI40:6	217.425	24.378	209.865	19.240	50.320	10.304	49.004	2.788	0.820	0.908
	**Total PI**	1298.301	68.772	1245.966	52.456	275.087	39.650	258.097	11.896	0.578	0.703
