# Supplementary material for: Spastin mutations impair coordination between lipid droplet dispersion and reticulum
Source: PLoS Genet. 2020 Apr 21;16(4):e1008665. doi: 10.1371/journal.pgen.1008665 (PMC7173978; doi:10.1371/journal.pgen.1008665)
Supplement: S2 Table — (PDF) [file pgen.1008665.s006.pdf]

Table S2: List of Primers

Method	Gene			Name	sequence 5' -> 3'	
	Name	Species	Accession#			
Cloning	Spastin	zebrafish	Q6NW58	ATTB1-zSpastin M61 For	For	ggggacaagtttgtacaaaaaagcaggcttcATGGCGGCCCGGGCCAAAGAGTG
				ATTB1-zSpastin M1 - For	For	ggggacaagtttgtacaaaaaagcaggcttcATGAATTCTGGGCACAAAGCGA
				ATTB2-zSpastin Rev	Rev	ggggaccactttgtacaagaaagctgggttTACGCCAGTCGTGTCTCCGTAC
	Spastin	human	Q9UBP0	ATTB1 Spastin M87 For	For	ggggacaagtttgtacaaaaaagcaggcttcATGGCAGCCAAGAGGAGCTCCGG
				ATTB1-hSpastin M1 For	For	ggggacaagtttgtacaaaaaagcaggcttcATGAATTCTCCGGGTGGACGAG
				ATTB2-hSpastin M1 Rev	Rev	ggggaccactttgtacaagaaagctgggttTTAAACAGTGGTATCTCCAAAG
	Seipin	human	Q96G97	ATTB1 Seipinα For	For	ggggacaagtttgtacaaaaaagcaggcttcATGGTCAACGACCCTCCAGTAC
				ATTB1 Seipinβ For	For	ggggacaagtttgtacaaaaaagcaggcttcATGTCTACAGAAAAGGTAGACC
				ATTB2 Seipin Rev	Rev	ggggaccactttgtacaagaaagctgggttGGAACTAGAGCAGGTGGGGCGC
	REEP1	human	Q9H902	ATTB1-REEP1 For	For	ggggacaagtttgtacaaaaaagcaggcttcATGGTGTCATGGATCATCTCCAG
				ATTB2-REEP1 Rev	Rev	ggggaccactttgtacaagaaagctgggttGGCGGTGCCTGAGCTGCTAGCGC
	Atlastin1	human	Q8WXF7	ATTB1-Atlastin1 For	For	ggggacaagtttgtacaaaaaagcaggcttcATGGCCAAGAACCGCAGGGACAG
				ATTB2-Atlastin1 Rev	Rev	ggggaccactttgtacaagaaagctgggttCATTTTTTTCTTTTCTGATTGTTCAG
	Spartin	human	Q8N0X7	ATTB1-hSpartin For	For	ggggacaagtttgtacaaaaaagcaggcttcATGGAGCAAGAGCCACAAAATGG
				ATTB2-hSpartin Rev	Rev	ggggaccactttgtacaagaaagctgggttTTTATCTTTCTTCTTTGCCTCC
qPCR	18s	zebrafish	KY486501	18s-F	For	AGCGTGCGGGAAACCACGAG
				18s-R	Rev	AAGCCGCAGGCTCCACTCCT
	Seipin	zebrafish	E7EYM9	Seipin-F	For	TCAGCAGAGACGAGAAGAAGCA
				Seipin-R	Rev	GAAGCGGATCTGAGAGTAGTTCACT
	Atlastin1	zebrafish	B8A6H1	Atlastin1-F	For	CAGAGAGGTGGTGGCCATTT
				Astlastin1-R	Rev	TTCACTGGCCTGGCTGTACA
	REEP1	zebrafish	E7F615	REEP1-F	For	TTGTGCTGGCTCCCATTCTAC
				REEP1-R	Rev	ATACAGCACACTGGATCCCTTTG
	SpartinB	zebrafish	A0A0R4ITC8	Spartin-F	For	CCACTGTAACCAAAAGCCTTCAT
				Spartin1-R	Rev	ACGTCCAACATGTCCAGCAA
rt PCR	Spastin	zebrafish	Q6NW58	Spastin M1 For	For	ATGAATTCTGGGCACAAAGCGAG
				Spastin M61 For	For	ATGGCGGCCCGGGCCAAAGAGTG
				Spastin Rev	Rev	TACGCCAGTCGTGTCTCCGTACT
				Spastin Ex3 For	For	GAAGGCAGACAGAGCCAGAAAA
				Spastin Ex5 rev	Rev	TGATTGTTTTGAGGGCCGGTCC
	XBP1	zebrafish	A7MCI6	xbp1-P5	For	CGAGACAAGACGAGTGATCTGCT
				xbp1-P2	Rev	GCAGGAGATCAGACTCAGAGTCTG
	GAPDH	zebrafish	Q5XJ10	GAPDH For	For	ACTTTGTCATCGTTGAAGGT
				GAPDH Rev	Rev	TGTCAGATCCACAACAGAGA
in situ hybridization	Spastin	zebrafish	Q6NW58	ISH T7 Spastin Fw Sense	For	gaaattaatacgactcactatagggATGAATTCTGGGCACAAAGCG
				ISH T7 Spastin Rw Anti-S	Rev	gaaattaatacgactcactatagggCTGGAGACTGTGAAAGCAGAT
				ISH Spastin Fw Anti-S	For	ATGAATTCTGGGCACAAAGCG
				ISH Spastin Rw Sense	Rev	CTGGAGACTGTGAAAGCAGAT

Cloning: Lower cases correspond to ATTB1/2 flanking sequences

ISH: Lower cases correspond to T7 promotor
