# Supplementary material for: Spastin mutations impair coordination between lipid droplet dispersion and reticulum
Source: PLoS Genet. 2020 Apr 21;16(4):e1008665. doi: 10.1371/journal.pgen.1008665 (PMC7173978; doi:10.1371/journal.pgen.1008665)
Supplement: S1 Text — Details of specific sections. (PDF) [file pgen.1008665.s007.pdf]

Spastin mutations impair coordination between lipid droplet dispersion and reticulum

Yoan Arribat, Dogan Grepper, Sylviane Lagarrigue, Timothy Qi, Sarah Cohen, Francesca Amati

SUPPORTING INFORMATION

MATERIALS AND METHODS

RNA extraction and qPCR

Total RNA was extracted from approximately 100 zebrafish embryos or larvae at the developmental stages specified in the text and from adult tissues (brain and skeletal muscle) using Trizol Reagent (15596026, Invitrogen, ThermoFischer Scientific, Waltham, MA, United States), following the procedure in Peterson S et al, 2009 [1]. cDNA was synthesized by reverse transcription (RT) using both Oligo(dT) (18418020, Invitrogen) and Random Hexamers (N8080127, Invitrogen) with Super Script II Reverse Transcriptase (18064014, Invitrogen) based on standard protocols. 1 µl of each RT product was mixed with Power SYBR Green PCR Master Mix (4367659, ThermoFischer Scientific), forward and reverse primers (Table S2) to a final volume of 10 µl. Reaction mix was cycled on StepOnePlus (Applied Biosystems, Foster City, CA). Relative expression of mRNA was estimated using the 2-ΔΔCT method using 18S rRNA as reference. qPCR primers were predicted with the program *Primer Express 3.0* and screened for an optimal efficiency.

RT-PCR targeting zebrafish Spastin variants and GAPDH were amplified using primers described in Table S2. PCR were performed with Go TaqMaster Mix (M4021, Promega, Madison, WI). XBP1 splice region was amplified using primers XBP1-P2 and XBP1-P5 (Table S2) as previously described [2].

Cells transfection and Immunolabeling

HeLa cells were grown in DMEM1X GlutaMAX medium (31966-021, GIBCO, ThermoFischer Scientific) supplemented with 10% FBS (10270-016, GIBCO) and penicillin/streptomycin. Transfection was performed with Lipofectamine 3000 (L3000-01, Invitrogen) following supplier instructions. Immunostaining procedure was performed 48h later.400µM of OA were added in DMEM/2% BSA for 18h. When indicated, 1µM of nocodazole (M1404, Sigma-Aldrich, St. Louis, MO) and 20µM of Taxol (Paclitaxel, P3456, Invitrogen) were added for 12h prior to apply the immunolabeling protocol. Cells were fixed 10min in PBS1X/PFA4% then permeabilized in PBX1X/Triton 0.25% for 5min. Primary antibodies were applied in 1% BSA blocking solution for 90min at room temperature: anti-αTubulin DM1A (T6199, Sigma-Aldrich, 1/1000); anti-acetylated Tubulin (T6793, Sigma-Aldrich, 1/500); anti-Tom20 (sc-17764, SCBT, 1/500); anti-LaminA/C (sc-20681, SCBT, 1/250); anti-Calreticulin-Alexa488 (ab196158, Abcam, 1/250); anti-Flag M2-FITC (F4049-.2mg, Sigma-Aldrich, 1/500).

Secondary antibody were diluted at 1/1000 and added for 1h: Alexa Fluor 647 Anti-mouse (A31571, Invitrogen), Alexa Fluor 488 Anti-mouse (A11029, Life technologies), Alexa Fluor® 488 AffiniPure Goat Anti-Rabbit (111-545-144, Jackson). Lipid droplet and nucleus counterstaining were performed as followed: BODIPY665/676 1mg/ml (B3932, Invitrogen, 1/1000, 30min), BODIPY493/503 1mg/ml (D3922, Invitrogen, 1/1000, 30min), Hoechst 10mg/ml (H3570, Invitrogen, 1/8000, 2min).

Multi spectral analysis

U2OS cells were cultured in 8-well chambered coverglass (Cellvis, Mountain View, CA) coated with 10 µg/ml fibronectin (Millipore, Burlingon, MA). Cells were prepared for multispectral imaging as previously described [3]-[4]. Briefly, cells were transfected to express LAMP1-CFP (lysosomes), mito-EGFP (mitochondria), ss-YFP-KDEL (ER), mOrange2-SKL (peroxisomes), mCherry-tagged spastin constructs, and then incubated with Bodipy 665/676 (50 ng/ml, Life Technologies) for 16 h to label LDs. Images were acquired on a Zeiss 880 laser confocal scanning microscope equipped with a 32-channel multi-anode spectral detector (Carl Zeiss, Oberkochen, Germany) using a 63x/1.4 NA objective lens, at 37 °C and 5% CO_2. All fluorophores were excited simultaneously using 458, 514 and 561, and 640 nm lasers, and images were collected onto a linear array of 32 photomultiplier tube elements in lambda mode at 9.7 nm bins. The emission spectra of the fluorophores were defined using images from singly-labeled cells, and images from multiply-labeled cells were subjected to linear unmixing using Zen software (Carl Zeiss).

For image analysis, we used a custom Matlab pipeline (AnalyzeMultispectral, available at: https://github.com/TimXQi/Cohen-Lab). This pipeline was used to generate cell and organelle masks through median or Gaussian blurring, Otsu thresholding, and watershed segmentation when appropriate. In channels containing larger organelles, objects below 10 square pixels were considered noise and filtered out. Organelle number and size were calculated from the corresponding organelle masks. ER and mitochondria area fraction were calculated by dividing the number of pixels in the organelle mask by the number of pixels in the cell mask. Contact area fraction was calculated by generating an overlap image between two distinct organelle masks, summing the number of pixels, and dividing by the number of pixels in the first organelle mask. All values reported are medians. Unpaired t-tests with Bonferroni correction were used to compare spastin-overexpressing cells with controls.

Lipidomics

Seven months old male zebrafish were anesthetized on ice and killed by decapitation. Brain and skeletal muscle were immediately dissected and flash frozen in liquid nitrogen. For free fatty acid methyl ester (FAMEs) analyses, lipids corresponding to around 3 mg of brain and 20 mg of muscle were extracted according to Bligh and Dyer [5] in dichloromethane/methanol/water (2.5:2.5:2.1, v/v/v), in the presence of the internal standards heptadecanoate acid (2µg). The

lipid extract was directly methylated in boron trifluoride methanol solution 14% (Sigma, 1ml) and heptane (1ml) at RT for 10min. After addition of water (1ml) to the crude extract, FAMEs were extracted with heptane (3ml), evaporated to dryness and dissolved in ethyl acetate (20µl). FAMEs (1µl) were analysed by gas-liquid chromatography [6] on a Clarus 600 Perkin Elmer system using a Famewax RESTEK fused silica capillary columns (30 m x 0.32 mm i.d, 0.25 µm film thickness). Oven temperature was programmed from 110°C to 220°C at a rate of 2°C per min and the carrier gas was hydrogen (0.5 bar). The injector and the detector were at 225°C and 245°C respectively.

For phospholipids, ceramides, sphingomyelins and neutral lipids, lipids corresponding to approximately 1.5 mg of brain and 10 mg of muscle were extracted adapted from Bligh and Dyer [5] in dichloromethane/methanol (2% acetic acid) / water (2,5:2,5:2 v/v/v), in the presence of the internals standards (Cer d18:1/15:0 16 ng; PE 12:0/12:0 180 ng ; PC 13:0/13:0 16ng ; SM d18:1/12:0 16 ng ; PI 16:0/17:0 30 ng ; PS 12:0/12:0 156.25 ng, 4 µg of stigmasterol, 4 µg of cholesteryl heptadecanoate and 8 µg of glyceryl trinonadecanoate). The solution was centrifuged at 1500 rpm for 3 min. The organic phase was collected and dried under azote, then dissolved in 20 µL of ethyl acetate for neutral lipid analysis and then in 50 µL in MeOH for main phospholipids and sphingolipids profiling. The extract was then stored at−20 ◦C prior to analysis. Main phospholipids and sphingolipids were profiled using an Agilent 1290 UPLC system coupled to a G6460 triple quadripole spectrometer (Agilent Technologies) and using MassHunter software for data acquisition and analysis. An Kinetex HILIC column (Phenomenex, 50 x 4,6 mm, 2,6µm) was used for LC separations. The column temperature was controlled at 40°C. The mobile phase A was Acetonitrile; and B was 10 mM ammonium formate in water at pH 3,2. The gradient was as follows: from 10% to 30% B in 10 min; 10-12 min, 100% B; and then back to 10% B at 13 min for 1 min re-equilibrium prior to the next injection. The flow rate of mobile phase was 0,3 mL/min and the injection volume was 5 µL. An electrospray source was employed in positive (for Cer, PE, PC and SM analysis) and negative ion mode (for PI and PS analysis). The collision gas was azote. Needle voltage was set at + 4000 V. Several scan modes were used. First, to obtain the naturally different specie's mass, we analyzed cells lipid extracts with a precursor ion scan of 184 m/z, 241 m/z and 264 m/z to PC/SM, PI and Cer respectively; and a neutral loss scan of 141 and 87 to PE and PS respectively. The collision energy optimums for Cer, PE, PC, SM, PI, PS were 25 eV, 20 eV, 30 eV, 25 eV, 45 eV, and 22 eV respectively. Then the corresponding SRM transitions were used in order to quantify different PL species for each class. Two MRM acquisitions are necessaries because important differences between PL classes. Datas were treated using QqQ Quantitative (vB.05.00) and Qualitative analysis software (vB.04.00).

For neutral lipid molecular species,1µl of the lipid extract was analysed by gas-liquid chromatography on a FOCUS Thermo Electron system using an Zebron-1 Phenomenex fused silica capillary columns (5mX0,32mm i.d, 0.50 µm film thickness)[7]. Oven temperature was

programmed from 200°C to 350°C at a rate of 5°C per min and the carrier gas was hydrogen (0.5 bar). The injector and the detector were at 315°C and 345°C respectively.

References

1. Peterson SM, Freeman JL. RNA isolation from embryonic zebrafish and cDNA synthesis for gene expression analysis. J Vis Exp. 2009(30). DOI: 10.3791/1470
2. Li J, Chen Z, Gao LY, Colorni A, Ucko M, Fang S, et al. A transgenic zebrafish model for monitoring xbp1 splicing and endoplasmic reticulum stress in vivo. Mech Dev. 2015;137:33-44. DOI: 10.1016/j.mod.2015.04.001
3. Valm AM, Cohen S, Legant WR, Melunis J, Hershberg U, Wait E, et al. Applying systems-level spectral imaging and analysis to reveal the organelle interactome. Nature. 2017;546(7656):162-7. DOI: 10.1038/nature22369
4. Cohen S, Valm AM, Lippincott-Schwartz J. Multispectral Live-Cell Imaging. Current protocols in cell biology. 2018;79(1):e46. DOI: 10.1002/cpcb.46
5. Bligh EG, Dyer WJ. A rapid method of total lipid extraction and purification. Can J Biochem Physiol. 1959;37(8):911-7. DOI: 10.1139/o59-099
6. Lillington JM, Trafford DJ, Makin HL. A rapid and simple method for the esterification of fatty acids and steroid carboxylic acids prior to gas-liquid chromatography. Clin Chim Acta. 1981;111(1):91-8. DOI: 10.1016/0009-8981(81)90425-3
7. Barrans A, Collet X, Barbaras R, Jaspard B, Manent J, Vieu C, et al. Hepatic lipase induces the formation of pre-beta 1 high density lipoprotein (HDL) from triacylglycerol-rich HDL2. A study comparing liver perfusion to in vitro incubation with lipases. J Biol Chem. 1994;269(15):11572-7
